# A frequency quantum interpretation of the surface renewal model of mass transfer

**DOI:** 10.1098/rsos.170103

**Published:** 2017-07-05

**Authors:** Chanchal Mondal, Siddharth G. Chatterjee

**Affiliations:** Department of Paper and Bioprocess Engineering, SUNY College of Environmental Science and Forestry, 1 Forestry Drive, Syracuse, NY, USA

**Keywords:** Danckwerts distribution, frequency quantum, Higbie distribution, mass transfer, surface renewal model, turbulence

## Abstract

The surface of a turbulent liquid is visualized as consisting of a large number of chaotic eddies or liquid elements. Assuming that surface elements of a particular age have renewal frequencies that are integral multiples of a fundamental frequency quantum, and further assuming that the renewal frequency distribution is of the Boltzmann type, performing a population balance for these elements leads to the Danckwerts surface age distribution. The basic quantum is what has been traditionally called the rate of surface renewal. The Higbie surface age distribution follows if the renewal frequency distribution of such elements is assumed to be continuous. Four age distributions, which reflect different start-up conditions of the absorption process, are then used to analyse transient physical gas absorption into a large volume of liquid, assuming negligible gas-side mass-transfer resistance. The first two are different versions of the Danckwerts model, the third one is based on the uniform and Higbie distributions, while the fourth one is a mixed distribution. For the four cases, theoretical expressions are derived for the rates of gas absorption and dissolved-gas transfer to the bulk liquid. Under transient conditions, these two rates are not equal and have an inverse relationship. However, with the progress of absorption towards steady state, they approach one another. Assuming steady-state conditions, the conventional one-parameter Danckwerts age distribution is generalized to a two-parameter age distribution. Like the two-parameter logarithmic normal distribution, this distribution can also capture the bell-shaped nature of the distribution of the ages of surface elements observed experimentally in air–sea gas and heat exchange. Estimates of the liquid-side mass-transfer coefficient made using these two distributions for the absorption of hydrogen and oxygen in water are very close to one another and are comparable to experimental values reported in the literature.

## 1. Introduction

At the turn of the twentieth century, physics was faced with the ‘ultraviolet catastrophe’. The classical Rayleigh–Jeans formula was unable to explain the bell-shaped nature of the observed spectrum of blackbody radiation. The problem was resolved by Max Planck, who thereby laid the foundation of quantum mechanics. Planck invoked the now celebrated assumption that the energy of a particle (e.g. a gas molecule in a box or radiation in a cavity) could not take arbitrary values but only discrete multiples of some fundamental energy. With this energy quantum concept, Planck was able to derive his famous formula for the energy density of blackbody radiation, which reduces to the Rayleigh–Jeans formula in the limit of low frequencies [1]. In 1951, Danckwerts [2] published his classic paper on gas absorption in a turbulent liquid in which he presented the surface renewal model of mass transfer. This model visualizes the gas–liquid interface, where the absorption occurs, to be continuously replenished by fresh liquid elements that arrive from the bulk liquid. In contrast with the earlier model of Higbie [3] which had assumed that all surface elements had the same residence time at the gas–liquid interface (i.e. uniform age distribution), Danckwerts derived an exponential age distribution by using the postulate that all surface elements, irrespective of their individual ages, had equal probability of being replaced by fresh elements arriving from the bulk liquid. The only parameter that appears in this distribution is the frequency or rate of surface renewal *S*, which is the mean rate of production of fresh surface according to Danckwerts [2], and which depends on the prevailing hydrodynamic conditions. This well-known distribution, which carries his name and an early experimental verification of which was provided by Lamb *et al.* [4] in the case of gas absorption into a stirred liquid, has since found wide use in chemical engineering, and over the years, many applications and extensions of the surface renewal model have appeared in the literature [5–17] (table 1).

**Table 1.** Nomenclature.

*a* = parameter of the generalized Danckwerts age distribution
*A* = parameter in equation (2.5)
*c*(*x*, *t*) = dissolved-gas concentration in a surface element at location *x* and time *t*, kmol m^−3^
*c*_b_ = dissolved-gas concentration in the bulk liquid, kmol m^−3^
*c*_s_ = dissolved-gas concentration at the gas–liquid interface, kmol m^−3^
*C*_1_─C_3_ = constants in equation (1.1)
*D* = diffusion coefficient of the dissolved gas in the liquid, m^2^ s^−1^
*E* = rate of energy dissipation per unit mass of fluid, W kg^−1^
*f*(*t*) = steady-state age distribution of surface elements, s^−1^
f*(t*) = dimensionless steady-state age distribution of surface elements ( = *f*(*t*)/*S*)
$f_{\mathrm{crit}}^{*}=f^{*}(t_{\mathrm{crit}}^{*})$
*f*(*t*, *t*_p_) = age distribution of surface elements at process time *t*_p_, s^−1^
*F*(*t, t*_p_) = cumulative age distribution of surface elements at process time *t*_p_
*k*_L_ = liquid-side mass-transfer coefficient, m s^−1^
*K* = constant given by equation (A.11) (in electronic supplementary material)
*K*_1_, *K*_2_ = constants in equation (A.5)
*m* = mean of the logarithmic normal distribution (equation (5.1))
*M*_s_ = modulus of surface elasticity of the liquid or fluid, kg s^−2^
*n* = constant given by equation (A.15)
*N* = 0, 1, 2, 3,…
*p* = root of equation (A.4)
*r* = ratio of the rate of dissolved-gas transfer to the bulk liquid to the rate of gas absorption at the gas–liquid interface
*R* = individual renewal frequency of surface elements (can take only integral multiples of *S*), s^−1^
$\bar{R}(t)$ = average renewal frequency of surface elements with an age of *t* (i.e. average growth rate of *F*(*t, t*_p_)), s^−1^
*R*_abs_ = steady-state rate of gas absorption, kmol (m^2^ s)^−1^
*R*_abs_(*t*_p_) = average rate of gas absorption at process time *t*_p_, kmol (m^2^ s)^−1^
*R*_inst_(*t*) = instantaneous rate of gas absorption in a surface element having an age of *t*, kmol (m^2^ s)^−1^
*R*_trans_ = steady-state rate of dissolved-gas transfer to the bulk liquid, kmol (m^2^ s)^−1^
*R*_trans_(*t*_p_) = net rate of transfer of dissolved gas to the bulk liquid at process time *t*_p_, kmol (m^2^ s)^−1^
$R_{\mathrm{abs}}^{*}(t_{p}^{*})$ = dimensionless age-averaged rate of gas absorption (equation (3.7))
$R_{\mathrm{trans}}^{*}(t_{p}^{*})$ = dimensionless age-averaged rate of transfer of dissolved gas to the bulk liquid (equation (3.8))
*S* = fundamental renewal frequency of surface elements, s^−1^
*t* = age of a surface element, s
t* = dimensionless age of a surface element (=*St*)
*t*_crit_ = value of age where *f*(*t*) is maximum, s
$t_{\mathrm{crit}}^{*}=St_{\mathrm{crit}}$
*t*_p_ = process time, s
*t*_ren_ = mean eddy renewal or burst time, s
$t_{p}^{*}$ = dimensionless process time (=*St*_p_)
*u*(*t*) = unit step function
*x* = distance into the liquid measured from the gas–liquid interface, m
*y* = parameter of Γ(*z*, *y*)
*z* = parameter of Γ(*z*, *y*)
*greek letters*
*α* = constant given by equation (A.17)
*Γ*(*z*, *y*) = extended Euler gamma function (defined by equation (3.10))
*δ*(*t*) = delta function, s^−1^
*λ* = variable of integration
*ν* = kinematic viscosity of liquid or fluid, m^2^ s^−1^
*ρ* = density of liquid or fluid, kg m^−3^
σ = standard deviation of logarithmic normal distribution
*τ* = hydrodynamic parameter in the Higbie model (i.e. Higbie time), s
*τ*_d_ = delay or lag parameter, s

In mass-transfer studies, the surface renewal model is considered to be more realistic than the film model because it predicts that the liquid-side mass-transfer coefficient *k*_L_ is proportional to the square root of the diffusion coefficient *D* of the dissolved gas in the liquid—a result that has often been confirmed experimentally. For example, Kuthan & Broz [18] obtained experimental values of *k*_L_ for the absorption of helium, nitrogen and propane by a liquid film of aqueous ethylene glycol flowing over a smooth wetted wall and an expanded metal sheet. For the case of the wetted wall, they found *k*_L_ ∝ *D*^0.5^, which is in agreement with the surface renewal model. For the expanded metal sheet, *k*_L_ ∝ *D*^0.64^ and the film-penetration model was found to be more applicable. According to Astarita [19], in the case of a liquid in contact with a solid or a more viscous liquid phase, *k*_L_ ∝ *D*^2/3^. All of this is in sharp contrast to the film model which predicts that *k*_L_ ∝ *D*.

The surface of a turbulent liquid is characterized by bursting and chaotic movements of eddies that emerge from beneath the surface, and by the presence of turbulent sweeps, upwellings, downwellings and vortices that profoundly influence the interfacial mass-transfer process [20–22]. According to Komori *et al.* [20], mass transfer across the gas–liquid interface is dominated by large-scale, surface renewal eddies with the liquid-side mass-transfer coefficient being proportional to the square root of the surface renewal frequency. And according to Banerjee [21], liquid-side controlled gas exchange in the case of clean, non-breaking interfaces is well predicted by surface renewal models with the renewal frequency being that of the turbulent sweeps that impinge on the interface. Metzger & Dobbins [23] presented the following theoretical expression for *S*:

1.1 $$S=\frac{C_{1}{C_{2}}^{3/4}{C_{3}}^{3}\rho \nu^{3/4}E^{3/4}}{M_{s}},$$

where *C*_1_–*C*_3_ are constants, *ρ* is the density of the fluid, *ν* is the kinematic viscosity of the fluid, *M*_s_ is the modulus of surface elasticity of the fluid and *E* is the specific rate of energy dissipation in the fluid as a whole (due to turbulent mixing). They deduced this relation by equating the resisting pressure (which opposes eddy motion) at the surface of the fluid to the product of the fluid density and the square of the eddy velocity, and by assuming that this velocity is proportional to the Kolomogoroff velocity factor (which is equal to the product of the kinematic viscosity of the fluid and the energy dissipation rate per unit mass of fluid raised to a power of 0.25). Equation (1.1) shows that there is a direct relation between *S* and *E*, and, keeping the random and erratic movements of the surface eddies in mind, the possibility arises that this motion is a result of the specific rate of energy dissipation in the fluid being quantized, which would be manifested in a quantization of the eddy renewal frequency. This raises the question of whether the Danckwerts age distribution has a deeper theoretical basis and can be derived by arguments similar to those used by Planck, with the surface renewal frequency *S* playing some kind of a fundamental role.

The answer to this question is provided in §2 of this manuscript. Specifically, the surface of a turbulent liquid is visualized as consisting of a large number of chaotic eddies or liquid elements. Assuming that surface elements of a particular age have renewal frequencies that are integral multiples of a fundamental frequency quantum, and further assuming that the renewal frequency distribution is of the Boltzmann type, performing a population balance for these elements leads to the Danckwerts surface age distribution. The basic quantum is what has been traditionally called the rate of surface renewal, *S*. Higbie surface age distribution follows if the renewal frequency distribution of such elements is assumed to be continuous. The development to be presented in what follows will lead to the derivation of four different unsteady-state age distributions. These four distributions correspond to four different hypotheses about the behaviour of liquid elements at the gas–liquid interface and reflect different initial states of the interface when the absorption commences. The first two age distributions (Cases 1 and 2) are two different versions of the Danckwerts model and the third one (Case 3) is based on the uniform and Higbie distributions, whereas the fourth (Case 4) is a mixed distribution. These distributions should be useful in modelling unsteady-state gas absorption in a gas–liquid reactor or a packed tower with the surface renewal model. Although these age distributions have appeared in previous and more recent works, as will be indicated later, none of them have traced their basis to the frequency quantum hypothesis that is proposed in this paper.

The frequency quantum hypothesis used herein is to be treated as an ansatz. The parameter *S*, which depends upon the level of turbulence or flow instability and fluid properties, is postulated to be a fundamental quantity which governs both the rate of gas absorption at the gas–liquid interface and the rate of dissolved-gas transfer to the bulk liquid from the interface. It is this basic parameter which determines the mean eddy renewal time—the relation between them is explored later.

A portion of the energy supplied for fluid mixing is transmitted to eddies of finer and finer scales (i.e. the Richardson cascade) and is ultimately dissipated by fluid friction. It may be speculated that another portion of this energy causes groups of fluid molecules to reach different excited states, and it is the transition of molecules between these states that leads to the release of discrete bursts of energy, which is reflected in the chaotic or discontinuous motion observed in turbulent flow. For example, Llaguno & Muriel [24] explained the multilevel turbulent data of the flow of nitrogen at room temperature obtained in a specially constructed experimental apparatus by postulating transitions between different rotational energy levels of the nitrogen molecule, which are caused by intermolecular collisions. By introducing a modification (postulated to arise from inelastic interactions of quantum origin among fluid molecules, which may be in different excited states) to the incompressible Navier–Stokes equation, Jirkovsky & Muriel [25] were able to derive the flattened paraboloid velocity profile in the turbulent flow of a fluid in a pipe and between two parallel plates, and also to fit the experimentally observed velocity profile with a single exponential-type equation in the near wall (viscous and buffer sublayers), intermediate and far away region in the turbulent flow of water in a channel. We note that the universal velocity profile for turbulent flow in a pipe is conventionally represented by three separate expressions depending upon the distance from the wall [26].

There is a vital aspect of the surface renewal model that has received scant attention in the literature where the focus has been on the rate of interphase transfer, i.e. the rate of gas absorption at the gas–liquid interface. Owing to surface renewal, there is a constant movement of liquid elements between the bulk liquid and the gas–liquid interface with a consequent net transfer of dissolved gas from the interface to the bulk liquid. In the case of physical absorption, as will be shown later, except at steady state, these two rates are not equal because of the accumulation of absorbed gas in the surface elements, while for absorption with chemical reaction, these two rates will not be equal even under steady-state conditions as demonstrated by Chatterjee [27] for the case of a first-order reaction. This is because of the consumption of dissolved gas by the chemical reaction in the surface elements. It should be noted that it is the rate of transfer of dissolved gas to the bulk liquid and not the rate of gas absorption at the gas–liquid interface that forms a part of the mass balance for dissolved gas in the bulk liquid, without which a rational design of gas–liquid contacting equipment is not possible. As pointed out by Chatterjee [27], this conceptual error has been made by some investigators and even by Danckwerts himself in Chapter 8 of his well-known book on gas–liquid reactions [28] in his general mass-balance equations for the dissolved-gas and liquid phase reactant in a packed column, although he presented an extensive discussion of this problem in the context of the film model in Chapter 6 of this book. To our knowledge, Loffler & Merchuk [29] and Merchuk [30], using the film penetration model of mass transfer, were the first to present the general form of the equation for the rate of dissolved-gas transfer for the case of absorption with a first-order reaction in a continuous flow, stirred tank reactor, assuming no gas phase resistance to mass transfer. The case of a finite gas-phase mass-transfer resistance was considered by Chatterjee [27] who, using the surface renewal and film penetration models, derived analytical expressions for the dissolved-gas transfer rate for the case of absorption with a first-order reaction under steady-state conditions. This development made possible an analysis with the surface renewal model of a gas–liquid reactor in which the dissolved gas is consumed by a first-order reaction [27], analysis of which has conventionally been made with the film model [28,31].

The use of the four age distributions derived in this work is illustrated in §3 of this manuscript where they are used in the theoretical modelling of transient physical absorption of a gas into a large volume of liquid (i.e. constant bulk liquid concentration of dissolved gas), assuming negligible gas-side mass-transfer resistance. In particular, explicit mathematical expressions for the transient rates of absorption of the gas at the gas–liquid interface and transfer of dissolved gas to the bulk liquid are derived. Section 4 of the manuscript presents numerical comparisons among the four age distribution cases.

Although experimental confirmation of the Danckwerts age distribution has been provided by Lamb *et al.* [4] for the case of a stirred liquid and by Lesage *et al.* [16] in the case of pipe flow, some studies by the physical oceanography community have reported that the age distribution of surface elements in air–sea heat and gas exchange, as measured both directly and indirectly, does not follow the exponential age distribution of Danckwerts but rather the logarithmic normal (LN) or Chi distributions [32–34]. This is addressed in §5, where it is shown that the generalized Danckwerts (GD) age distribution (derived in the electronic supplementary material, S1), which follows from an extension of the conventional Danckwerts age distribution, gives a shape which is very close to that of the LN distribution. Using both of these distributions, estimates of the liquid-side mass-transfer coefficient in the case of the absorption of hydrogen and oxygen in water at three different wind speeds, which were used by Garbe *et al.* [32] in their experiments, are presented. Section 6 of the manuscript offers some concluding remarks.

We note that the case of transient physical gas absorption in which the gas-phase resistance is finite and the bulk liquid concentration of the dissolved gas is a function of process time is a more difficult theoretical problem. For such a situation, equations for the rates of gas absorption and dissolved-gas transfer can be expressed in the form of convolution integrals; these have been provided by Chatterjee [35] using the age distribution for Case 1. However, only general conclusions were inferred and no numerical results were presented by him.

## 2. Theoretical development

### 2.1. Population balance

The surface (assumed to be of unit area) of a turbulent liquid is visualized as being populated by a mosaic of chaotic eddies or liquid elements. At any time *t*_p_ since the start of the process (e.g. absorption of a gas in a well-mixed liquid, crossflow membrane filtration, etc.), such elements are assumed to have different surface ages, which will range from zero to *t*_p_. Let *F*(*t*, *t*_p_) be the cumulative fraction of surface elements that have ages between 0 and *t* at process (or ‘clock’) time *t*_p_ and let $\bar{R}(t)$ be the average growth rate of this cumulative fraction. Performing a population balance at process time *t*_p_ for elements with ages lying within the time interval from *t* to *t* + d*t* then gives

2.1 $$\frac{\partial F(t,t_{p})}{\partial t}=F(t,t_{p})\bar{R}(t).$$

Integrating equation (2.1) yields

2.2 $$F(t,t_{p})=\int_{0}^{t}F(\lambda ,t_{p})\bar{R}(\lambda )\;d\lambda .$$

For reasons that will become clear shortly, we will refer to $\bar{R}(t)$, which has dimensions of inverse time, as the *average* renewal frequency of surface elements having ages lying in the time interval from *t* to *t* + d*t*.

### 2.2. Expression for $\bar{R}(t)$

$\bar{R}(t)$ is taken to be a weighted average of the *individual* renewal frequencies *R* of surface elements. This quantity, which is a reflection of the energy content of individual or groups of surface elements, is assumed to come in discrete packets (due to the random, discontinuous motions of the elements) which are integral multiples of a basic or *fundamental* frequency quantum. This basic quantum, as mentioned before, is taken to be equal to what has been conventionally called the rate of renewal of liquid elements at the surface, which is denoted by *S*. Thus, *R* = 0, *S*, 2*S*, 3*S*, …. If we now assume a Boltzmann-type distribution for the individual renewal frequency *R*, the average renewal frequency $\bar{R}(t)$ of elements with an age of *t* at the interface is given by

2.3 $$\bar{R}(t)=\frac{\sum_{N=0}^{\infty }Re^{-Rt}}{\sum_{N=0}^{\infty }e^{-Rt}}=\frac{\sum_{N=0}^{\infty }NSe^{-NSt}}{\sum_{N=0}^{\infty }e^{-NSt}}=\frac{Se^{-St}/{\left(1-e^{-St}\right)}^{2}}{1/(1-e^{-St})}=\frac{Se^{-St}}{1-e^{-St}}.$$

According to equation (2.3), $\bar{R}(t\to 0)=1/t$ and $\bar{R}(t\to \infty )=0$, i.e. younger surface elements have a greater average renewal rate, which is in accord with physical intuition.

In the theoretical development up to this point, as one conceptually moves from *S* (fundamental frequency quantum) through *R* (individual renewal frequency) to $\bar{R}(t)$ (average renewal frequency), one journeys from the essence to the appearance.

### 2.3. General expression for *F*(*t*, *t*_p_)

Substituting equation (2.3) into equation (2.2) yields

2.4 $$F(t,t_{p})=\int_{0}^{t}F(\lambda ,t_{p})\frac{Se^{-S\lambda }}{1-e^{-S\lambda }}\;d\lambda ,$$

whose solution is

2.5 $$F(t,t_{p})=A(1-e^{-St}),$$

where *A* is a parameter whose value depends upon the initial state of the surface when the process was started at *t*_p_ = 0. Equation (2.5) contains the essence of the age distributions derived in this work.

Differentiating equation (2.5) with respect to *t* yields the density age distribution *f*(*t*,*t*_p_), i.e.

2.6 $$f(t,t_{p})=\frac{\partial F(t,t_{p})}{\partial t}=ASe^{-St}.$$

From equation (2.6), it can be shown that

2.7 $$\frac{\partial f(t,t_{p})}{\partial t}=-Sf(t,t_{p}).$$

In the conventional derivation of the density age distribution *f*, e.g. that given by Danckwerts [2], equation (2.7) is the starting point and the cumulative age distribution *F* can then be obtained by integrating the density age distribution *f* with respect to *t*. However, in this method, the essential nature of the parameter *S*, that it is a fundamental frequency quantum, is obscured. This is in contrast with the procedure adopted in our work in which equation (2.1) for the cumulative age distribution *F* is the starting point whose time derivative is the density age distribution *f*.

In the development that follows, we will consider the four age distributions mentioned previously. The first two are unsteady-state versions of the Danckwerts (steady-state) model. Both have appeared previously in the literature and, as will be shown subsequently, reduce to the famous steady-state age distribution proposed by Danckwerts as the process time *t*_p_ → ∞.

#### 2.3.1. Case 1 (Danckwerts distribution)

The surface (assumed to be of unit area, as mentioned earlier) is instantaneously and completely formed at *t*_p_ = 0 with liquid elements flowing into it from the bulk liquid and departing from it to the bulk liquid at a constant rate for *t*_p_ ≥ 0. As

2.8 $$F(t_{p},t_{p})=1,$$

the value of *A* in equation (2.5) is given by

2.9 $$A=\frac{1}{1-e^{-St_{p}}},$$

which transforms equation (2.5) into

2.10 $$F(t,t_{p})=\frac{1-e^{-St}}{1-e^{-St_{p}}}\;\mathrm{for}\;0\leq t\leq t_{p}.$$

Differentiating equation (2.10) with respect to *t* yields the following age distribution:

2.11 $$f(t,t_{p})=\frac{\partial F(t,t_{p})}{\partial t}=\frac{Se^{-St}}{1-e^{-St_{p}}}\;\mathrm{for}\;0\leq t\leq t_{p}.$$

The above equation has been used in the modelling of crossflow ultrafiltration and microfiltration [36–40]. As *t*_p_ → ∞, it reduces to the well-known, steady-state age distribution *S*e^−*St*^, which was proposed by Danckwerts [2].

#### 2.3.2. Case 2 (Danckwerts distribution)

In this case, it is assumed that there are liquid elements, visualized as being ‘blue’, that are already present on the surface at *t*_p_ = 0 when the process starts and ‘red’ elements begin displacing the blue elements by the mechanism of surface renewal. At any time *t*_p_, the surface will therefore consist of a mixture of both red and blue elements, the population of the latter decreasing as *t*_p_ increases. The red elements will have ages lying within 0 ≤ *t* < *t*_p_ while the blue elements will have ages exactly equal to *t*_p._ From this mental picture and equations (2.5) and (2.8), it follows that

2.12 A=1

and

2.13 $$F(t,t_{p})=\left\{ \begin{array}{ll} 1-e^{-St} & \mathrm{for}\;0\leq t<t_{p},\\ 1 & \mathrm{at}\;t=t_{p}. \end{array}\right.$$

The above equation has a discontinuity at *t* = *t*_p_ that becomes vanishingly small as *t*_p_ → ∞. This discontinuity is due to the fraction of blue elements present on the surface at process time *t*_p_, which is equal to $e^{-St_{p}}$. Equation (2.13) can be written as

2.14 $$F(t,t_{p})=1-e^{-St}+u(t-t_{p})e^{-St}\;\mathrm{for}\;0\leq t\leq t_{p},$$

where *u*(*t*) is the unit step function. Differentiating this equation with respect to *t* yields

2.15 $$f(t,t_{p})=Se^{-St}[1-u(t-t_{p})]+\delta (t-t_{p})e^{-St}\;\mathrm{for}\;0\leq t\leq t_{p},$$

where *δ*(*t*) is the delta function.

Chung *et al.* [8] and Sada *et al.* [9] have previously presented equation (2.15) while a derivation of this equation, based on a stochastic population balance of interfacial fluid elements, has been provided by Fan *et al.* [14]. We note that, in the general equation for the unsteady-state age distribution provided by Chung *et al.* [8] of which equation (2.15) is a special case, the form of the steady-state age distribution has to be known or assumed *a priori* in order to derive the specific form of the unsteady-state age distribution, unlike in this work. In a recent paper, Zhang & Chatterjee [41] used this age distribution in the derivation of analytical expressions for the permeate flux decline and cake build-up in constant pressure, crossflow microfiltration. It is once again observed that, as *t*_p_ → ∞, equation (2.15) reduces to the steady-state Danckwerts age distribution.

#### 2.3.3. Case 3 (uniform and Higbie distributions)

We again assume a Boltzmann-type distribution for *R* but now assume that this distribution is continuous. The average renewal frequency $\bar{R}(t)$ of elements that have a residence time of *t* at the surface is obtained from

2.16 $$\bar{R}(t)=\frac{\int_{0}^{\infty }Re^{-Rt}dR}{\int_{0}^{\infty }e^{-Rt}dR}=\frac{1}{t}.$$

Substituting equation (2.16) into equation (2.2) yields the following equation:

2.17 $$F(t,t_{p})=\int_{0}^{t}F(\lambda ,t_{p})\frac{1}{\lambda }d\lambda ,$$

whose solution, which satisfies equation (2.8), is

2.18 $$F(t,t_{p})=\frac{t}{t_{p}}\;\mathrm{for}\;0\leq t\leq t_{p},\;0<t_{p}\leq \tau .$$

Differentiating equation (2.18) with respect to *t* yields the uniform age distribution:

2.19 $$f(t,t_{p})=\frac{1}{t_{p}}\;\mathrm{for}\;0\leq t\leq t_{p},\;0<t_{p}\leq \tau .$$

The age distribution represented by equation (2.19) corresponds to the situation when the surface (initially empty) is ‘filling up’ with liquid elements that arrive from the bulk liquid, and is being formed; i.e. there is no outflow of elements from the surface back to the bulk liquid until a certain value of *t*_p_ = *τ* (Higbie time). Once the surface is completely formed at *t*_p_ = *τ*, liquid elements which flow into it from the bulk liquid also start departing from it to the bulk liquid at a constant rate. If it is assumed that thereafter (i.e. for *t*_p_ > *τ*) all liquid elements have the same residence time at the surface with elements flowing into and out of the surface in a *plug-flow* fashion, equation (2.19) reduces to the Higbie distribution:

2.20 $$\begin{array}{rl} f(t,t_{p}) & =\frac{1}{\tau }\;\mathrm{for}\;0\leq t\leq \tau ,\;t_{p}>\tau ,\\ & =0\;\mathrm{for}\;t>\tau ,\;t_{p}>\tau , \end{array}\}$$

where *τ* is the residence time of liquid elements at the interface, which is a hydrodynamic parameter. Thus, the Higbie distribution (equation (2.20)) is a special case of the uniform distribution (equation (2.19)).

It can be observed that, as *St*_p_ → 0, i.e. as *S* → 0 (low surface renewal) or as *t*_p_ → 0 (near the start of the process), which implies that *St* → 0, equation (2.11) reduces to equation (2.19), i.e. the Danckwerts distribution (Case 1) reduces to the uniform distribution. This is analogous to the reduction of the Planck formula to the classical Rayleigh–Jeans formula in the limit of low frequencies in the energy spectrum radiated by a blackbody [1].

#### 2.3.4. Case 4 (mixed distribution)

This distribution can be thought of as being a combination of ideas contained in the distributions discussed earlier, and has also been used by Zhang & Chatterjee [41] to model permeate flux decline and cake build-up in constant pressure, crossflow microfiltration. For the benefit of the reader, we recapitulate their derivation. Initially, i.e. at *t*_p_ = 0, the surface is assumed to be empty of liquid elements. At *t*_p_ = 0+ when the process starts, the surface starts filling up with such elements, with no outflow of elements to the bulk liquid until the surface renewal mechanism is assumed to trigger at *t*_p_ = 1/*S* after which fresh liquid elements from the bulk begin to displace those already occupying the surface, which start flowing to the bulk liquid. The age distribution can be derived as follows.

Within the time interval 0 < *t*_p_ ≤ 1/*S*, the age distribution of the surface elements will be uniform and given by equation (2.19). Thus

2.21 $$\int_{0}^{t_{p}}f(t,t_{p})\;dt=\int_{0}^{t_{p}}\frac{1}{t_{p}}\;dt=1.$$

When surface renewal begins at *t*_p_ = 1/*S*, let all liquid elements that already occupy the surface be thought of as being blue, while those elements from the bulk that start displacing the blue elements from *t*_p_ = 1/*S* onwards be thought of as having the colour red. Similar to Case 2 of the Danckwerts distribution, at any time *t*_p_ > 1/*S* the surface will consist of both red and blue elements with ages lying from 0 to *t*_p_ − 1/*S* and *t*_p_ − 1/*S* to *t*_p_, respectively. The age distribution for the red elements, which have ages in 0 ≤ *t* ≤ *t*_p_ − 1/*S*, will be given by *S*e^−*St*^ (see equation (2.15)), while that for the blue elements, which have ages in *t*_p_ − 1/*S* < *t* ≤ *t*_p_, will be equal to $Se^{1-St_{p}}$ because (see equation (2.8))

2.22 $$F(t_{p},t_{p})=\int_{0}^{t_{p}}f(t,t_{p})dt=\int_{0}^{t_{p}-1/S}Se^{-St}dt+\int_{t_{p}-1/S}^{t_{p}}Se^{1-St_{p}}dt=1.$$

Therefore, it follows that the age distribution is given by

2.23 $$f(t,t_{p})=\{\begin{array}{llll} \frac{1}{t_{p}} & \mathrm{for} & 0\leq t\leq t_{p}, & 0<t_{p}\leq \frac{1}{S},\\ Se^{-St} & \mathrm{for} & 0\leq t\leq t_{p}-\frac{1}{S}, & t_{p}>\frac{1}{S},\\ Se^{1-St_{p}} & \mathrm{for} & t_{p}-\frac{1}{S}<t\leq t_{p}, & t_{p}>\frac{1}{S}. \end{array}$$

The cumulative age distribution corresponding to equation (2.23) is expressed by

2.24 $$F(t,t_{p})=\{\begin{array}{llll} \frac{t}{t_{p}} & \mathrm{for} & 0\leq t\leq t_{p}, & 0<t_{p}\leq \frac{1}{S},\\ 1-e^{-St} & \mathrm{for} & 0\leq t\leq t_{p}-\frac{1}{S}, & t_{p}>\frac{1}{S},\\ 1+Se^{1-St_{p}}(t-t_{p}) & \mathrm{for} & t_{p}-\frac{1}{S}<t\leq t_{p}, & t_{p}>\frac{1}{S}. \end{array}$$

## 3. Unsteady-state gas absorption in a large volume of liquid

As an example of the application of the different age distributions, consider the physical, unsteady-state absorption of a gas into a large volume of liquid (i.e. constant bulk liquid concentration of dissolved gas). If the gas-side mass-transfer resistance is negligible, the concentration profile and instantaneous rate of absorption of the gas per unit area in a surface element (of infinite depth) having an age of *t* are given by [19,28]

3.1 $$\frac{c(x,t)-c_{b}}{c_{s}-c_{b}}=\mathrm{erfc}\left(\frac{x}{2\sqrt{Dt}}\right)$$

and

3.2 $$R_{\mathrm{inst}}(t)=(c_{s}-c_{b})\sqrt{\frac{D}{\pi t}},$$

where *c*(*x*,*t*) is the dissolved-gas concentration in the element at location *x* (measured from the gas–liquid interface) and time *t*, *c*_s_ and *c*_b_ are the dissolved-gas surface and bulk liquid concentrations (assumed to be constants), respectively, and *D*, as mentioned earlier, is the diffusion coefficient of the dissolved gas in the liquid. The average rate of absorption of the gas at process time *t*_p_ [*R*_abs_(*t*_p_)] can then be obtained from

3.3 $$R_{\mathrm{abs}}(t_{p})=\int_{0}^{t_{p}}R_{\mathrm{inst}}(t)f(t,t_{p})dt,$$

We will now present an expression for the transient net rate of transfer *R*_trans_(*t*_p_) of the dissolved gas to the bulk liquid. The expression for *R*_trans_ for surface elements of infinite depth is given by

3.4 $$R_{\mathrm{trans}}(t_{p})=S\int_{0}^{t_{p}}\int_{0}^{\infty }[c(x,t)-c_{b}]\;f(t,t_{p})dx\;dt,$$

where the first and second terms on the right-hand side of equation (3.4) represent the convective transfer (due to the surface renewal mechanism) of dissolved gas to the bulk liquid from the gas–liquid interface and that from the bulk liquid to the interface, respectively.

For later use, we define the following dimensionless quantities:

3.5 $$\begin{array}{rl} t^{*} & =St, \end{array}$$

3.6 $$\begin{array}{rl} t_{p}^{*} & =St_{p}, \end{array}$$

3.7 $$\begin{array}{rl} R_{\mathrm{abs}}^{*}(t_{p}^{*}) & =\frac{R_{\mathrm{abs}}(t_{p})}{\sqrt{DS}(c_{s}-c_{b})}, \end{array}$$

3.8 $$\begin{array}{rl} R_{\mathrm{trans}}^{*}(t_{p}^{*}) & =\frac{R_{\mathrm{trans}}(t_{p})}{\sqrt{DS}(c_{s}-c_{b})} \end{array}$$

3.9 $$\begin{array}{rl} r & =\frac{R_{\mathrm{trans}}(t_{p})}{R_{\mathrm{abs}}(t_{p})}. \end{array}$$

Also

3.10 $$\Gamma (z,y)=\int_{y}^{\infty }\lambda^{z-1}e^{-\lambda }d\lambda$$

and

3.11 $$\int_{0}^{\infty }\mathrm{erfc}\left(\frac{x}{2\sqrt{Dt}}\right)dx=2\sqrt{\frac{Dt}{\pi }},$$

where Γ(*z*, *y*) is the extended Euler gamma function.

### 3.1. Case 1 (Danckwerts distribution)

Using equation (2.11) and equations (3.1)–(3.11) yields the following:

3.12 $$\begin{array}{rl} R_{\mathrm{abs}}^{*}(t_{p}^{*}) & =\frac{\mathrm{erf}\sqrt{t_{p}^{*}}}{1-e^{-t_{p}^{*}}}, \end{array}$$

3.13 $$\begin{array}{rl} R_{\mathrm{trans}}^{*}(t_{p}^{*}) & =\frac{1}{1-e^{-t_{p}^{*}}}\left[1-\frac{2}{\sqrt{\pi }}\Gamma \left(\frac{3}{2},t_{p}^{*}\right)\right] \end{array}$$

3.14 $$\begin{array}{rl} \;\text{and}\;r(t_{p}^{*}) & =\frac{1}{\mathrm{erf}\sqrt{t_{p}^{*}}}\left[1-\frac{2}{\sqrt{\pi }}\Gamma \left(\frac{3}{2},t_{p}^{*}\right)\right]. \end{array}$$

Equations (3.12) and (3.13) are mathematically similar to the expressions for permeate flux decline and cake mass build-up, respectively, in constant pressure, crossflow microfiltration [42]. It is further seen from these equations that $R_{\mathrm{trans}}^{*}(t_{p}^{*})\neq R_{\mathrm{abs}}^{*}(t_{p}^{*})$ in general. Only as $t_{p}^{*}\to \infty$, both of these rates approach one another and reduce to

3.15 $$R_{\mathrm{abs}}^{*}(t_{p}^{*}\to \infty )\approx R_{\mathrm{trans}}^{*}(t_{p}^{*}\to \infty )=1,$$

which implies that $r(t_{p}^{*}\to \infty )=1$, i.e.

3.16 $$R_{\mathrm{abs}}(t_{p}^{}\to \infty )\approx R_{\mathrm{trans}}(t_{p}^{}\to \infty )=\sqrt{\mathrm{DS}}(c_{s}-c_{b}),$$

which is the well-known steady-state form of the absorption rate for the surface renewal model [28]. For small values of $t_{p}^{*}$, i.e. as $t_{p}^{*}\to 0$, equation (3.12) reduces to

3.17 $$R_{\mathrm{abs}}^{*}(t_{p}^{*}\to 0)=\frac{2}{\sqrt{\pi t_{p}^{*}}}.$$

It can also be shown that

3.18 $$r(t_{p}^{*}\to 0)=\frac{2}{3}t_{p}^{*}.$$

The condition $t_{p}^{*}\to 0$, i.e. *St*_p_ → 0 implies that *S* → 0 (low surface renewal) or *t*_p_ → 0 (near the start of absorption). Under either of these situations, equation (3.18) shows that the ratio of the rates of transfer to absorption varies linearly with the dimensionless process time $t_{p}^{*}$ with a slope of 2/3. From equations (3.17) and (3.18) it follows that

3.19 $$R_{\mathrm{trans}}^{*}(t_{p}^{*}\to 0)=\frac{4}{3\sqrt{\pi }}\sqrt{t_{p}^{*}}.$$

Thus, for $t_{p}^{*}\to 0$, the rate of gas absorption is inversely proportional to $\sqrt{t_{p}^{*}}$, whereas the rate of dissolved-gas transfer is directly proportional to it, i.e. they have an inverse relationship.

### 3.2. Case 2 (Danckwerts distribution)

From equation (2.15) and equations (3.1)–(3.11), it can be shown that

3.20 $$\begin{array}{rl} R_{\mathrm{abs}}^{*}(t_{p}^{*}) & =\mathrm{erf}\sqrt{t_{p}^{*}}+\frac{e^{-t_{p}^{*}}}{\sqrt{\pi t_{p}^{*}}}, \end{array}$$

3.21 $$\begin{array}{rl} R_{\mathrm{trans}}^{*}(t_{p}^{*}) & =1+\frac{2}{\sqrt{\pi }}\left[\sqrt{t_{p}^{*}}e^{-t_{p}^{*}}-\Gamma \left(\frac{3}{2},t_{p}^{*}\right)\right] \end{array}$$

3.22 $$\begin{array}{rl} \;\text{and}\;r(t_{p}^{*}) & =\frac{\sqrt{\pi }+2\left[\sqrt{t_{p}^{*}}e^{-t_{p}^{*}}-\Gamma (3/2,t_{p}^{*})\right]}{\sqrt{\pi }\mathrm{erf}\sqrt{t_{p}^{*}}+e^{-t_{p}^{*}}/\sqrt{t_{p}^{*}}}. \end{array}$$

Equation (3.20) has been presented before by Chung *et al.* [8] who used it to derive an expression for the time-averaged mass-transfer coefficient. They found good agreement of this expression with experimental data on mass transfer in liquid films in wetted wall columns and obtained values of *S*, which increases with the liquid mass flow rate, varying from 0.294 to 2.42 s^−1^ as the gas–liquid contact time decreased from 3.4 to 0.41 s. As $t_{p}^{*}\to \infty$, equations (3.20) and (3.21) reduce to equation (3.15) (i.e. $r(t_{p}^{*}\to \infty )=1$), whereas for $t_{p}^{*}\to 0$, equation (3.20) approaches the limit

3.23 $$R_{\mathrm{abs}}^{*}(t_{p}^{*}\to 0)=\frac{1}{\sqrt{\pi t_{p}^{*}}},$$

which is half of the rate for Case 1 (see equation (3.17)). Also, it can be shown that

3.24 $$r(t_{p}^{*}\to 0)=2t_{p}^{*},$$

which can be compared to equation (3.18). From equations (3.23) and (3.24), it follows that

3.25 $$R_{\mathrm{trans}}^{*}(t_{p}^{*}\to 0)=\frac{2}{\sqrt{\pi }}\sqrt{t_{p}^{*}},$$

which can be compared to equation (3.19).

### 3.3. Case 3 (uniform and Higbie distributions)

For this case, there are two time periods which have to be considered separately; these are 0 < *t*_p_ ≤ *τ* and *t*_p_ > *τ*. During the first period, the surface is being formed by the arrival of fresh liquid elements from the bulk liquid with no outflow of such elements back to the bulk liquid (i.e. *R*_trans_(*t*_p_) = 0). The surface is completely formed at *t*_p_ = *τ* after which, as indicated earlier, liquid elements flow to it from the bulk liquid and depart from it to the bulk liquid at a constant rate, with all elements residing on the surface for the same amount of time *τ*. Thus, *S* = 1/*τ*. For 0 < *t*_p_ ≤ *τ*, we use equations (2.19), (3.2), (3.3) and (3.5)–(3.9) to obtain

3.26 $$\begin{array}{rl} R_{\mathrm{abs}}^{*}(t_{p}^{*}) & =\frac{2}{\sqrt{\pi t_{p}^{*}}}\;\mathrm{for}\;0<t_{p}^{*}\leq 1, \end{array}$$

3.27 $$\begin{array}{rl} R_{\mathrm{trans}}^{*}(t_{p}^{*}) & =0\;\mathrm{for}\;0<t_{p}^{*}\leq 1 \end{array}$$

3.28 $$\begin{array}{rl} \;\text{and}\;r(t_{p}^{*}) & =0\;\mathrm{for}\;0<t_{p}^{*}\leq 1. \end{array}$$

For *t*_p_ > *τ*, the age distribution, which is given by equation (2.20), is independent of *t*_p_ and the dimensionless absorption rate can be obtained by setting $t_{p}^{*}=1$ in equation (3.26), i.e.

3.29 $$R_{\mathrm{abs}}^{*}(t_{p}^{*})=\frac{2}{\sqrt{\pi }}\;\mathrm{for}\;t_{p}^{*}>1.$$

The above equation is the well-known expression for the absorption rate for the penetration or Higbie model [28]. The rate of dissolved-gas transfer has to be obtained from a modified form of equation (3.4) because, for *t*_p_ > *τ*, all elements reside on the surface for the same amount of time *τ*. This rate is given by

3.30 $$R_{\mathrm{trans}}(t_{p})=S\int_{0}^{t_{p}}\int_{0}^{\infty }[c(x,t)-c_{b}]dx\delta (t-\tau )dt=\frac{1}{\tau }\int_{0}^{\infty }[c(x,\tau )-c_{b}]dx.$$

As *τ* = 1/*S*, equation (3.30), after using equations (3.1) and (3.8), is transformed into

3.31 $$R_{\mathrm{trans}}^{*}(t_{p}^{*})=\frac{2}{\sqrt{\pi }}\;\mathrm{for}\;t_{p}^{*}>1.$$

From equations (3.9), (3.29) and (3.31), we obtain

3.32 $$r(t_{p}^{*})=1\;\mathrm{for}\;t_{p}^{*}>1.$$

It is observed from equations (3.28) and (3.32) that *r* undergoes a step change from 0 to 1 at $t_{p}^{*}=1$ (i.e. at *t*_p_ = *τ*) and thereafter remains constant at this value.

### 3.4. Case 4 (mixed distribution)

From equation (2.23) and equations (3.1)–(3.11), it can be deduced that

3.33 $$\begin{array}{rl} R_{\mathrm{abs}}^{*}(t_{p}^{*}) & =\frac{2}{\sqrt{\pi t_{p}^{*}}}\;\mathrm{for}\;0<t_{p}^{*}\leq 1, \end{array}$$

3.34 $$\begin{array}{rl} R_{\mathrm{trans}}^{*}(t_{p}^{*}) & =0\;\mathrm{for}\;0<t_{p}^{*}\leq 1, \end{array}$$

3.35 $$\begin{array}{rl} r(t_{p}^{*}) & =0\;\mathrm{for}\;0<t_{p}^{*}\leq 1 \end{array}$$

and

3.36 $$\begin{array}{rl} R_{\mathrm{abs}}^{*}(t_{p}^{*}) & =\mathrm{erf}\left(\sqrt{t_{p}^{*}-1}\right)+\frac{2}{\sqrt{\pi }}e^{1-t_{p}^{*}}\left[\sqrt{t_{p}^{*}}-\sqrt{t_{p}^{*}-1}\right]\;\mathrm{for}\;t_{p}^{*}>1, \end{array}$$

3.37 $$\begin{array}{rl} R_{\mathrm{trans}}^{*}(t_{p}^{*}) & =\frac{2}{\sqrt{\pi }}\left[\frac{\sqrt{\pi }}{2}-\Gamma \left(\frac{3}{2},t_{p}^{*}-1\right)+\frac{2}{3}e^{1-t_{p}^{*}}\left\{t{{\;}_{p}^{*}}^{3/2}-{\left(t_{p}^{*}-1\right)}^{3/2}\right\}\right]\;\mathrm{for}\;t_{p}^{*}>1 \end{array}$$

3.38 $$\begin{array}{rl} r(t_{p}^{*}) & =\frac{\sqrt{\pi }/2-\Gamma (3/2,t_{p}^{*}-1)+(2/3)e^{1-t_{p}^{*}}\left\{t{{\;}_{p}^{*}}^{3/2}-{\left(t_{p}^{*}-1\right)}^{3/2}\right\}}{(\sqrt{\pi }/2)\mathrm{erf}\left(\sqrt{t_{p}^{*}-1}\right)+e^{1-t_{p}^{*}}\left[\sqrt{t_{p}^{*}}-\sqrt{t_{p}^{*}-1}\right]}\;\mathrm{for}\;t_{p}^{*}>1. \end{array}$$

We note from equations (3.35) and (3.38) that *r* undergoes a sudden transition from 0 to 2/3 at $t_{p}^{*}=1$. Thereafter, as $t_{p}^{*}\to \infty$, *r* asymptotically approaches a value of 1.

## 4. Numerical comparison among the four different age distribution cases

Table 2 presents a summary of the results for the four cases that correspond to the different age distributions.

**Table 2.** Expressions for the dimensionless gas absorption and dissolved-gas transfer rates corresponding to the four different age distributions.

cases	age distribution *f*(*t*,t_p_)	dimensionless absorption rate $R_{\mathrm{abs}}^{*}(t_{p}^{*})$	dimensionless transfer rate $R_{\mathrm{trans}}^{*}(t_{p}^{*})$
1	$\frac{Se^{-St}}{1-e^{-St_{p}}}\;\mathrm{for}\;0\leq t\leq t_{p}$	$\mathrm{erf}\sqrt{t_{p}^{*}}/(1-e^{-t_{p}^{*}})$	$\frac{2}{\sqrt{\pi }(1-e^{-t_{p}^{*}})}\left[\frac{\sqrt{\pi }}{2}-\Gamma \left(\frac{3}{2},t_{p}^{*}\right)\right]$
2	$Se^{-St}[1-u(t-t_{p})]+\delta (t-t_{p})e^{-St}$	$\mathrm{erf}\sqrt{t_{p}^{*}}+\frac{e^{-t_{p}^{*}}}{\sqrt{\pi t_{p}^{*}}}$	1+AAAAA2π[tp∗e−tp∗−Γ(32,tp∗)]
	$\;\mathrm{for}\;0\leq t\leq t_{p}$		
3	AAA1tpfor0≤t≤tp, $\;0<t_{p}\leq \tau$	$\frac{2}{\sqrt{\pi t_{p}^{*}}}\;\mathrm{for}\;0<t_{p}^{*}\leq 1$	0
	$\frac{1}{\tau }\;\mathrm{for}\;0\leq t\leq \tau ,\;t_{p}>\tau$		
	$0\;\mathrm{for}\;t>\tau ,\;t_{p}>\tau$	$\frac{2}{\sqrt{\pi }}\;\mathrm{for}\;t_{p}^{*}>1$	$\frac{2}{\sqrt{\pi }}\;\mathrm{for}\;t_{p}^{*}>1$
4	AAA1tpfor0≤t≤tp,0<tp≤1S	$\frac{2}{\sqrt{\pi t_{p}^{*}}}\;\mathrm{for}\;0<t_{p}^{*}\leq 1$	0
	$Se^{-St}\;\mathrm{for}\;0\leq t\leq t_{p}-\frac{1}{S},\;t_{p}>\frac{1}{S}$		
	$Se^{1-St_{p}}\;\mathrm{for}\;t_{p}-\frac{1}{S}<t\leq t_{p},\;t_{p}>\frac{1}{S}$		
		$\mathrm{erf}\left(\sqrt{t_{p}^{*}-1}\right)+\frac{2}{\sqrt{\pi }}e^{1-t_{p}^{*}}$	$\frac{2}{\sqrt{\pi }}[\frac{\sqrt{\pi }}{2}-\Gamma \left(\frac{3}{2},t_{p}^{*}-1\right)$
		$\;\times \left[\sqrt{t_{p}^{*}}-\sqrt{t_{p}^{*}-1}\right]$	$\;+\frac{2}{3}e^{1-t_{p}^{*}}\left\{t{{\;}_{p}^{*}}^{3/2}-{\left(t_{p}^{*}-1\right)}^{3/2}\right\}]$
		$\;\mathrm{for}\;t_{p}^{*}>1$	$\;\mathrm{for}\;t_{p}^{*}>1$

**Figure 1.**
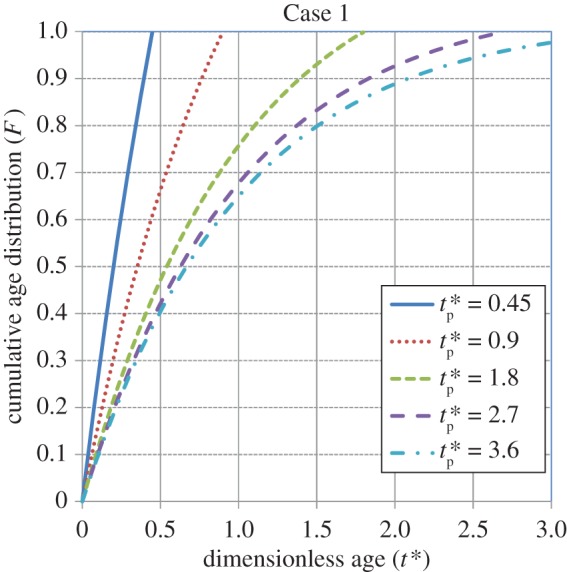
Cumulative age distribution for Case 1 (equation (2.10)). From Zhang & Chatterjee [41].

**Figure 2.**
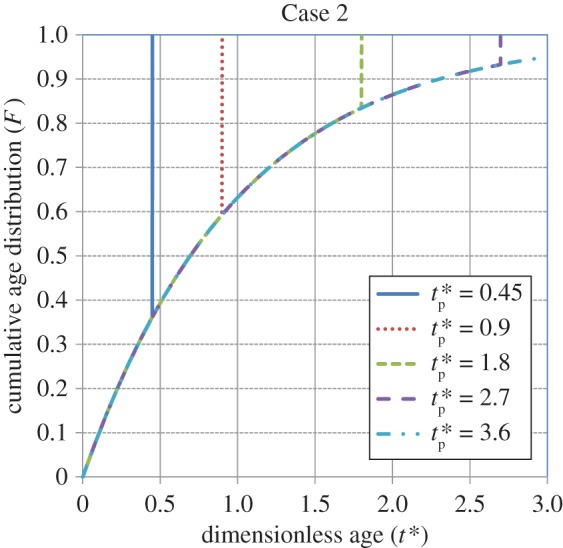
Cumulative age distribution for Case 2 (equation (2.13)). From Zhang & Chatterjee [41].

**Figure 3.**
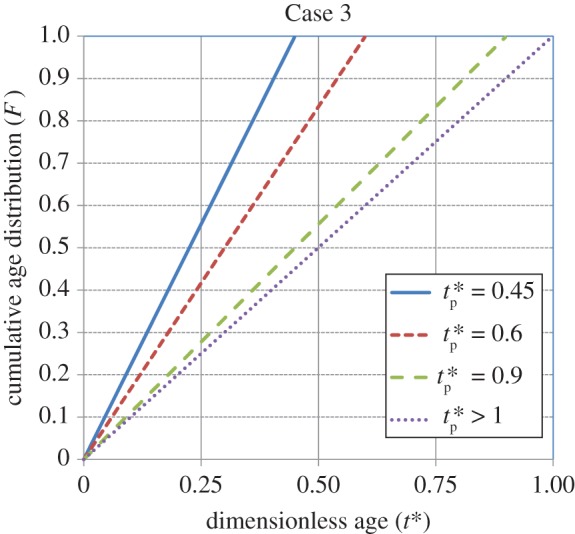
Cumulative age distribution for Case 3 (equation (2.18)). The distribution does not change beyond a value of $t_{p}^{*}=1$ (Higbie model).

**Figure 4.**
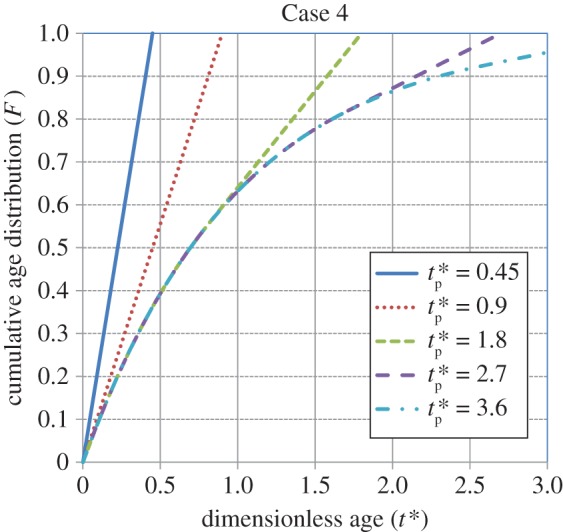
Cumulative age distribution for Case 4 (equation (2.24)). From Zhang & Chatterjee [41].

Figures 1–4 show the cumulative age distributions (equations (2.10), (2.13), (2.18) and (2.24)) as functions of the dimensionless age t*( = *St*) and dimensionless process time $t_{p}^{*}(=St_{p})$. Tables 3 and 4 report numerical values of the cumulative age distributions and the dimensionless (age-averaged) rates of gas absorption and dissolved-gas transfer to the bulk liquid at values of $t_{p}^{*}=0.9$ and 1.8, respectively, for the four cases.

**Table 3.** Numerical magnitudes of the cumulative age distribution and dimensionless rates of gas absorption and transfer when the dimensionless process time equals 0.9.

	cumulative age distribution		
cases	$F(t^{*}=0.6,t_{p}^{*}=0.9)$	$R_{\mathrm{abs}}^{*}(t_{p}^{*})$	$R_{\mathrm{trans}}^{*}(t_{p}^{*})$
1	0.76	1.38	0.65
2	0.45	1.06	0.82
3	0.67	1.19	0
4	0.67	1.19	0

**Table 4.** Numerical magnitudes of the cumulative age distribution and dimensionless rates of gas absorption and transfer when the dimensionless process time equals 1.8.

	cumulative age distribution	cumulative age distribution		
** **cases	$F(t^{*}=0.6,t_{p}^{*}=1.8)$	$F(t^{*}=0.9,t_{p}^{*}=1.8)$	$R_{\mathrm{abs}}^{*}(t_{p}^{*})$	$R_{\mathrm{trans}}^{*}(t_{p}^{*})$
1	0.54	0.71	1.13	0.83
2	0.45	0.59	1.01	0.94
3	0.33	0.5	1.13	1.13
4	0.45	0.6	1.02	0.92

It is observed from table 3 ($t_{p}^{*}=0.9$) that, at t* = 0.6, the values of *F* = 0.76, 0.45, 0.67 and 0.67 for Cases 1, 2, 3 and 4, respectively. Thus, the population of younger elements at the gas–liquid interface is greatest for Case 1, intermediate for Cases 3 and 4, and smallest for Case 2. As younger elements have a higher absorption rate (equation (3.2)) than older elements, the age-averaged absorption rate will be greatest for Case 1, intermediate for Cases 3 and 4, and lowest for Case 2. By contrast, younger elements will have a lower dissolved-gas concentration (equation (3.1)); therefore, the rate of dissolved-gas transfer to the bulk liquid will have an inverse relation to the rate of gas absorption at the gas–liquid interface. Thus, as illustrated in table 3, the average rate of transfer is higher for Case 2 compared to that for Case 1. The rate of transfer is zero for Cases 3 and 4 because the interface has not yet been completely filled up with liquid elements.

Table 4 shows the values of *F*, $R_{\mathrm{abs}}^{*}(t_{p}^{*})$ and $R_{\mathrm{trans}}^{*}(t_{p}^{*})$ at a higher value of $t_{p}^{*}=1.8$ for the four cases. At t* = 0.6, the population of younger elements at the gas–liquid interface is greatest for Case 1, intermediate for Cases 2 and 4, and smallest for Case 3. At t* = 0.9, the population of younger elements decreases in the order of cases 1, 4, 2 and 3, respectively. Not considering Case 3, which is an outlier because of the (imposed) sudden transition from a uniform distribution to the Higbie distribution at *t*_p _= *τ* (i.e. $t_{p}^{*}=1$), it is seen that the average rate of absorption is highest for Case 1, intermediate for Case 4 and lowest for Case 2. By contrast, the average transfer rate of dissolved gas to the bulk liquid is lowest for Case 1, intermediate for Case 4 and highest for Case 2. In summary, under unsteady-state conditions, the higher the rate of gas absorption at the gas–liquid interface, the lower is the transfer rate of dissolved gas to the bulk liquid. This conclusion will be reinforced by calculations for the rates of absorption and dissolved-gas transfer, which are presented shortly.

As $t_{p}^{*}$ increases, the initial state of the interface becomes relatively unimportant and *F* for Cases 1, 2 and 4 approaches the cumulative, steady-state age distribution:

4.1 $$F(t^{*},t_{p}^{*}\to \infty )=1-e^{-t^{*}}.$$

**Figure 5.**
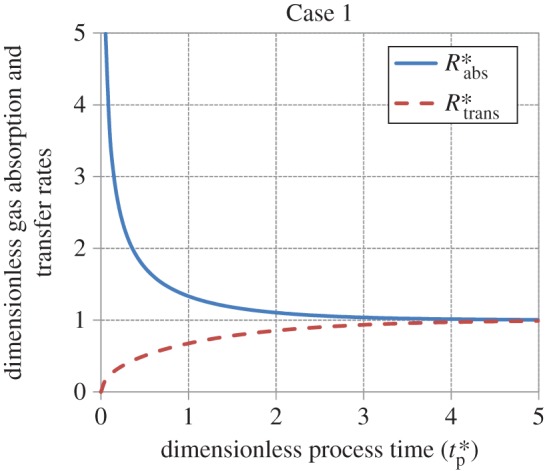
Behaviour of the rates of gas absorption and transfer as a function of process time in dimensionless coordinates for Case 1 (equations (3.12) and (3.13)).

**Figure 6.**
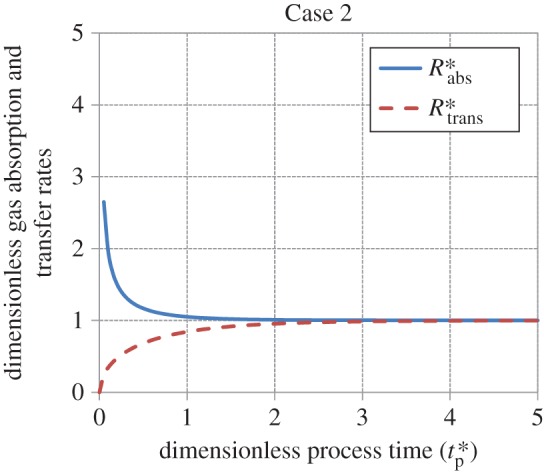
Behaviour of the rates of gas absorption and transfer as a function of process time in dimensionless coordinates for Case 2 (equations (3.20) and (3.21)).

**Figure 7.**
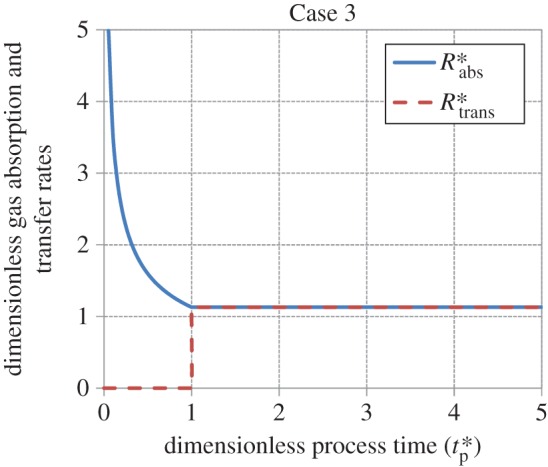
Behaviour of the rates of gas absorption and transfer as a function of process time in dimensionless coordinates for Case 3 (equations (3.26), (3.29), (3.27) and (3.31)).

**Figure 8.**
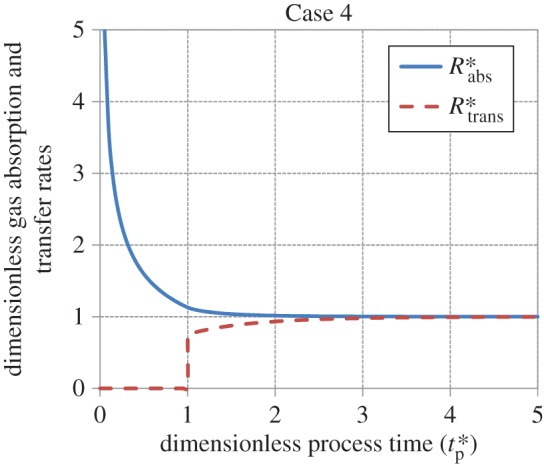
Behaviour of the rates of gas absorption and transfer as a function of process time in dimensionless coordinates for Case 4 (equations (3.33), (3.36), (3.34) and (3.37)).

Figures 5–8 show the age-averaged rates of gas absorption and dissolved-gas transfer to the bulk liquid as a function of process time in dimensionless coordinates. For the cases analysed in this work, the rate of absorption shows an exponential decay-like behaviour, decreasing from an initially high value to an asymptotic steady-state value as $t_{p}^{*}$ increases. This asymptotic value is 1 for Cases 1, 2 and 4, while it is 1.13 for Case 3 (equation (3.29)). The rate of transfer increases smoothly from a value of 0 to a value of 1 as $t_{p}^{*}\to \infty$ (i.e. as steady state is approached) for Cases 1 and 2. For Case 3, it is zero for values of $t_{p}^{*}\leq 1$ (equation (3.27)), increases in a stepwise fashion to 1.13 (equation (3.31)) at $t_{p}^{*}=1$ and thereafter remains constant at this value as $t_{p}^{*}\to \infty$. For Case 4, the rate of transfer is zero for values of $t_{p}^{*}\leq 1$ (equation (3.34)), i.e. when the interface is filling up with liquid elements, increases abruptly to 0.75 at $t_{p}^{*}=1$ and thereafter asymptotically approaches 1 as $t_{p}^{*}\to \infty$.

**Figure 9.**
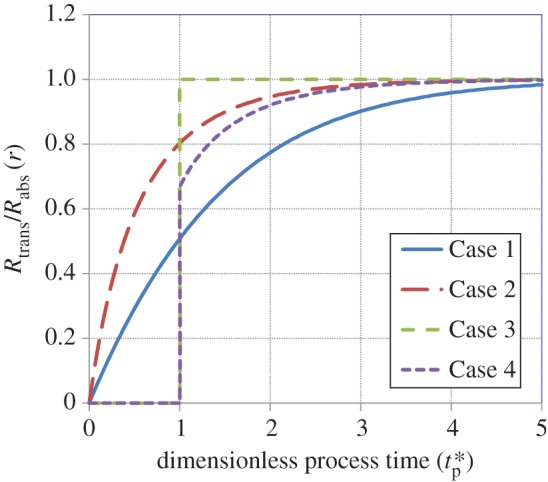
Ratio of the rate of gas transfer to that of gas absorption as a function of the dimensionless process time for the various cases (equations (3.14), (3.22), (3.28), (3.32), (3.35) and (3.38)).

Figure 9 presents the behaviour of the ratio *r* of the rate of transfer to that of absorption as a function of $t_{p}^{*}$. For all cases, *r* = 0 at $t_{p}^{*}=0$, and for Cases 1 and 2, it smoothly attains a value of 1 as $t_{p}^{*}\to \infty$. As indicated earlier, the rate of increase of *r* near the origin is much greater for Case 2 when compared with Case 1 (see equations (3.24) and (3.18)). Also, *r* is in general considerably smaller for Case 1 compared with Case 2, with the difference becoming vanishingly small as $t_{p}^{*}\to \infty$. This implies that there is a faster approach to steady state for Case 2 when compared with Case 1, which can also be observed in figures 5 and 6. For Case 3, *r* = 0 for $t_{p}^{*}\leq 1$ (equation (3.28)), increases in a stepwise fashion to 1 at $t_{p}^{*}=1$ (equation (3.32)) and thereafter remains constant at this value as $t_{p}^{*}\to \infty$. For Case 4, *r* = 0 for $t_{p}^{*}\leq 1$ (equation (3.35)), undergoes a sudden transition to 0.67 at $t_{p}^{*}=1$ and thereafter smoothly approaches a value of 1 as the process evolves towards steady state.

## 5. Comparison of the logarithmic normal and generalized Danckwerts age distributions

For simplicity, we will assume steady-state conditions (i.e. $t_{p}^{*}\to \infty$) in this section. It was mentioned earlier that, for air–sea gas and heat exchange, the LN distribution has been found experimentally to better represent the age distribution of surface elements than the exponential age distribution of Danckwerts. The LN distribution is given by

5.1 $$f(t)=\frac{1}{\sqrt{\pi }}\frac{1}{\sigma t}\exp\left[-{\left(\frac{\ln\;t-m}{\sigma }\right)}^{2}\right],$$

where the two parameters *m* and *σ* are the mean and standard deviation of the distribution. The mean eddy renewal or burst time *t*_ren_ can be obtained from

5.2 $$t_{\mathrm{ren}}=\int_{0}^{\infty }tf(t)dt.$$

By substituting equation (5.1) into equation (5.2), it can be shown that

5.3 $$t_{\mathrm{ren}}=\exp\left(m+\frac{\sigma^{2}}{4}\right).$$

The GD age distribution, derived in the electronic supplementary material, S1, is an extension of the conventional Danckwerts age distribution. The extension follows from the introduction of a time lag into the basic differential equation that describes the conventional Danckwerts model (see equation (A.2) in electronic supplementary material). We note that the time delay concept has been used in the modelling of various phenomena such as biogas production by anaerobic digestion of organic wastes [43,44], population dynamics, forest fires and wavefronts in reaction–diffusion systems [45,46], and circadian rhythms [47]. The GD age distribution is given by $f(t)=S({\left(2a+1\right)}^{a+1})/(\Gamma (a+1)){\left(St\right)}^{a}e^{-(2a+1)St},$ where *a* is a parameter of the distribution with the other parameter being *S*. We note that age distributions similar to equation (A.18) that involve the gamma function have been previously proposed by Harriott [6] and Bullin & Dukler [48]. However, these authors did not provide any theoretical justification, as done in the present work. We see that, for *a* = 0, equation (A.18) reduces to the standard or conventional Danckwerts age distribution, i.e. *f*(*t*) = *S*e^−*St*^. Substitution of equation (A.18) into equation (5.2) yields

5.4 $$t_{\mathrm{ren}}=\frac{1}{S}\left(\frac{a+1}{2a+1}\right).$$

For *a* = 0 (i.e. the standard Danckwerts age distribution), equation (5.4) gives *t*_ren_ = 1/*S*, i.e. the average eddy renewal time *t*_ren_ is equal to the reciprocal of the fundamental frequency quantum *S*, which is a well-known result. However, this is not true for other values of *a*. As the parameter *a* → ∞, *t*_ren_ → 0.5/*S*; thus for finite values of *a*, 0.5/S < *t*_ren_ < 1/*S* for the GD distribution.

**Figure 10.**
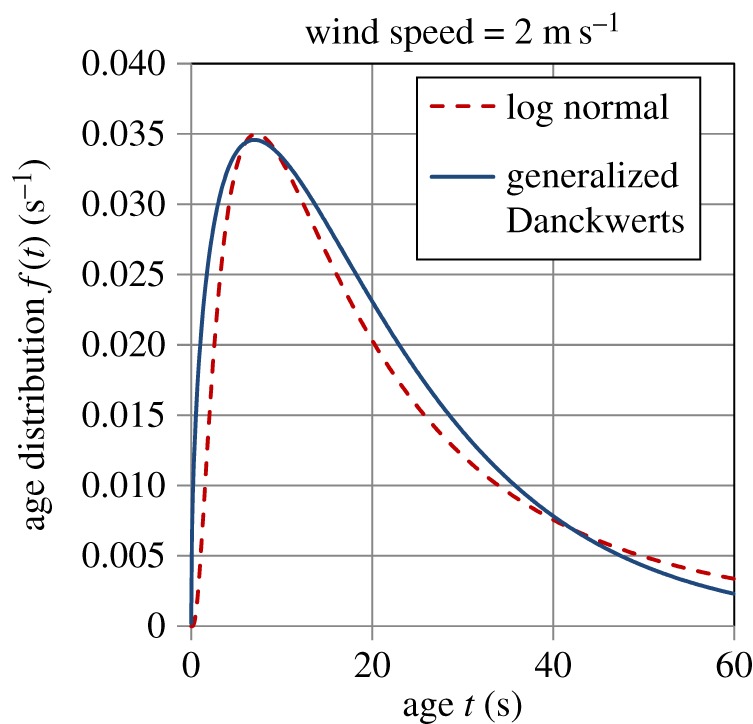
Comparison of the logarithmic normal (equation (5.1)) and generalized Danckwerts (equation (A.18)) age distributions for a wind speed of 2 m s^−1^. Parameter values are given in tables 5 and 6.

**Figure 11.**
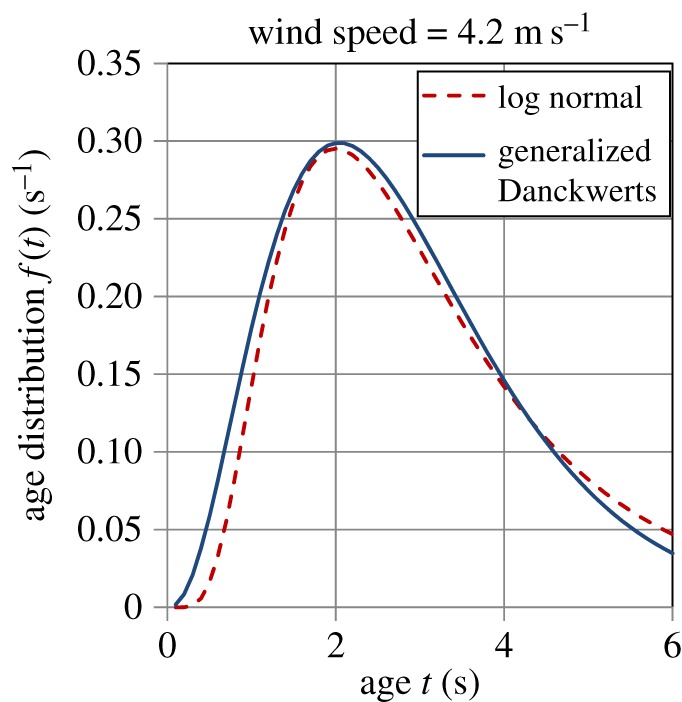
Comparison of the logarithmic normal (equation (5.1)) and generalized Danckwerts (equation (A.18)) age distributions for a wind speed of 4.2 m s^−1^. Parameter values are given in tables 5 and 6.

**Figure 12.**
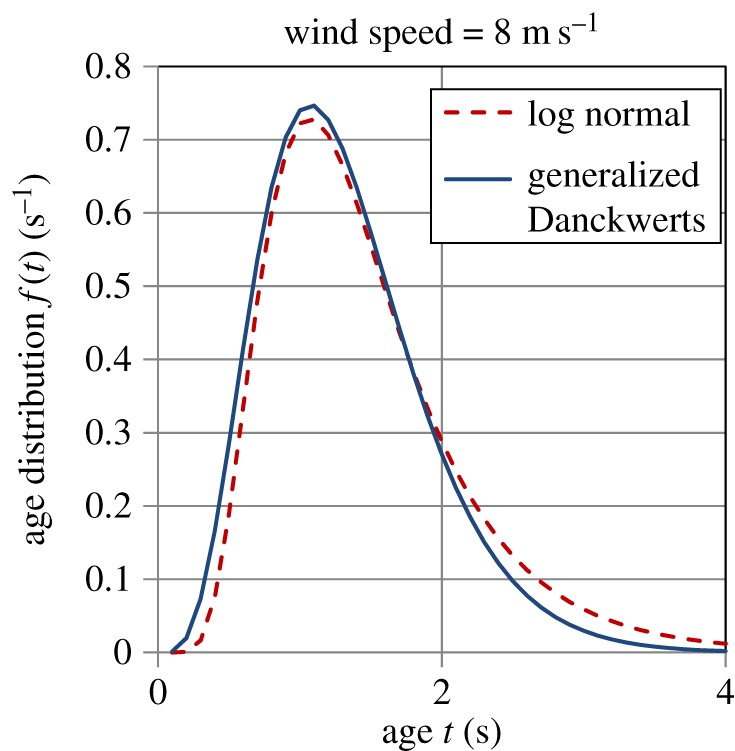
Comparison of the logarithmic normal (equation (5.1)) and generalized Danckwerts (equation (A.18)) age distributions for a wind speed of 8 m s^−1^. Parameter values are given in tables 5 and 6.

Garbe *et al*. [32] experimentally measured the age distribution of surface renewal events on the Heidelberg Aeolotron in air–water heat exchange. They found a good fit of the LN distribution (equation (5.1)) to these data and reported values of *m*, *σ* and *t*_ren_ (calculated by equation (5.3)) at wind speeds of 2, 4.2 and 8 m s^−1^; the values of these quantities are given in table 5. Figures 10–12 show plots of the LN age distribution using these values of *m* and *σ* for the three different wind speeds. The figures also show the GD age distribution (equation (A.18)), with the values of *a*, *S* and *t*_ren_ (calculated by equation (5.4)) given in table 6. It is evident that the LN and GD age distributions match one another quite closely. For the LN distribution, as the wind speed increases, the values of *m, σ* and *t*_ren_ decrease (table 5), whereas for the GD distribution, *a* and *S* increase, while *t*_ren_ decreases with wind speed (table 6). As the wind speed increases by a factor of 4, the mean eddy renewal or burst time decreases by a factor of 21 (LN distribution) and 16 (GD distribution), revealing the dramatic effect of turbulence. There is also a significant narrowing in the distribution of ages with a rise in wind speed as can be observed by comparing figures 10–12 among each other (see also in electronic supplementary material, figure A1 ).

**Table 5.** Values of the parameters of the logarithmic normal age distribution (equation (5.1)) for the experiments of Garbe *et al*. [32].

wind speed (m s^−1^)	*m*	*σ*	*t*_ren_ (s) (from equation (5.3))
2.0	2.934 ± 0.026	1.386 ± 0.026	30.38 ± 0.88
4.2	1.021 ± 0.011	0.812 ± 0.014	3.27 ± 0.04
8.0	0.277 ± 0.009	0.652 ± 0.012	1.47 ± 0.01

**Table 6.** Values of the parameters of the generalized Danckwerts age distribution (this work; equation (A.18)) for the experiments of Garbe *et al*. [32].

wind speed (m s^−1^)	*a*	*S* (s^−1^)	*t*_ren_ (s) (from equation (5.4))
2.0	0.5	0.036	21
4.2	2.5	0.204	2.86
8.0	4.2	0.417	1.33

The liquid side mass transfer coefficient *k*_L_ for the GD distribution is given by (see equations (A.16) and (A.17) in electronic supplementary material)

5.5 $$k_{L}=\frac{\Gamma (a+1/2)}{\Gamma (a+1)}\sqrt{\frac{(2a+1)DS}{\pi }}.$$

For *a* = 0, the above equation reduces to $k_{L}=\sqrt{DS}$, which is a well-known result of the conventional Danckwerts surface renewal model. For the LN distribution, it may be shown by using equations (3.2) and (5.1) that

5.6 $$k_{L}=\exp\left(\frac{\sigma^{2}}{16}\right)\sqrt{\frac{D}{\pi e^{m}}}.$$

Hutchinson & Sherwood [49] investigated the absorption of eight different pure gases at 25°C in a stirred flask containing water whose surface was exposed to the gas. Table 7 shows values of the liquid-side mass-transfer coefficient *k*_L_, which they obtained from dissolved-gas concentration measurements, for hydrogen and oxygen at two different stirrer speeds. This table also gives values of *D* (diffusion coefficient of dissolved gas in the liquid) that we estimated from values of the diffusion coefficient of the dissolved gas in water at 20°C reported in table III of [49] and by using the Wilke–Chang correlation [50] to correct them to 25°C. As the stirrer speed increases from 171 r.p.m. (revolutions per minute) to 1025 r.p.m., *k*_L_ varies from 1.06 × 10^−5^ to 3.25 × 10^−5^ m s^−1^ for hydrogen and 8.33 × 10^−6^ to 2.12 × 10^−5^ m s^−1^ for oxygen. Thus, a sixfold increase in the stirrer speed increases the value of *k*_L_ by a factor of 3.1 for hydrogen and 2.5 for oxygen.

**Table 7.** Data for the absorption of hydrogen and oxygen in a stirred flask containing water with the gas exposed above the stirred surface at 25°C. Values of the liquid-side mass-transfer coefficient (*k*_L_) and diffusion coefficient (*D*) were derived from the paper of Hutchinson & Sherwood [49] as explained in the text.

type of gas	r.p.m.	
*hydrogen*	171	1025
*k*_L_ (m s^−1^)	1.06 × 10^−5^	3.25 × 10^−5^
*D* (m^2^ s^−1^)	6.00 × 10^−9^	6.00 × 10^−9^
*oxygen*	171	1025
*k*_L_ (m s^−1^)	8.33 × 10^−6^	2.12 × 10^−5^
*D* (m^2^ s^−1^)	2.12 × 10^−9^	2.12 × 10^−9^

**Figure 13.**
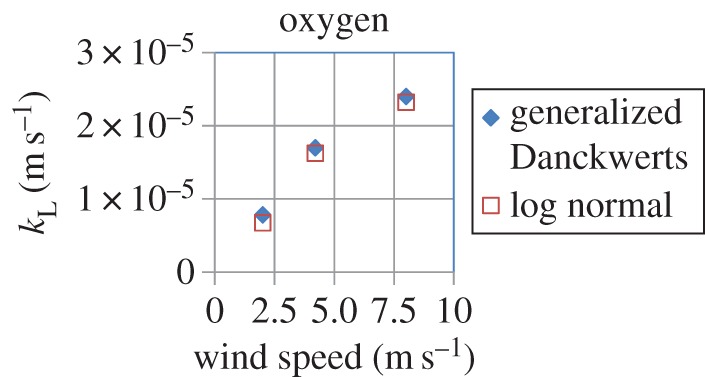
Calculated values of the liquid-side mass transfer coefficient *k*_L_ for oxygen absorption in water using the generalized Danckwerts (equation (5.5)) and logarithmic normal (equation (5.6)) models at the three different wind speeds used by Garbe *et al*. [32] in their experiments. Parameter values required for the calculations are given in tables 5–7.

**Figure 14.**
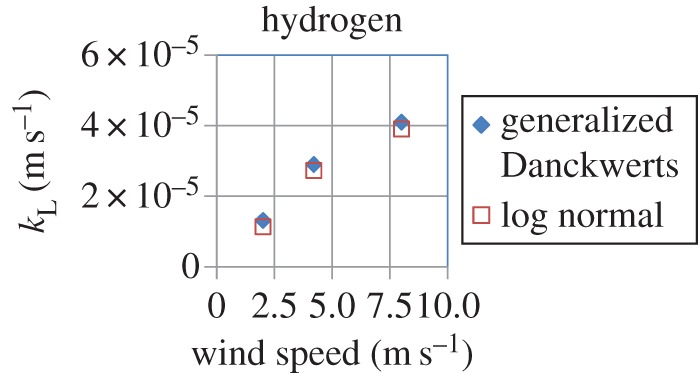
Calculated values of the liquid-side mass-transfer coefficient *k*_L_ for hydrogen absorption in water using the generalized Danckwerts (equation (5.5)) and logarithmic normal (equation (5.6)) models at the three different wind speeds used by Garbe *et al*. [32] in their experiments. Parameter values required for the calculations are given in tables 5–7.

Using the values of the diffusion coefficients of hydrogen and oxygen in water at 25°C (table 7), we calculated values of *k*_L_ for both the GD and LN distributions using equations (5.5) and (5.6), respectively, for the three different wind speeds used by Garbe *et al*. [32] in their experiments. The values of the parameters of the LN distribution (*m* and *σ*) and GD distribution (*a* and *S*) required for the calculations were obtained from tables 5 and 6, respectively. The results, shown in table 8 and figures 13 and 14, clearly demonstrate that both distributions predict very similar values of *k*_L_ at each wind speed, which, moreover, are comparable to those reported by Hutchinson & Sherwood [49] as can be observed from table 7. A fourfold increase in wind speed increases the value of *k*_L_ (for both gases) by factors of 3.4 and 3.1 for the LN and GD distributions, respectively. We also note that the increase in *k*_L_ with wind speed is not linear.

**Table 8.** Values of the liquid-side mass-transfer coefficient (*k*_L_) calculated by the logarithmic normal and generalized Danckwerts models at the three different wind speeds used by Garbe *et al*. [32]. Parameter values for these distributions were obtained from tables 5 and 6, whereas values of the diffusion coefficient of hydrogen and oxygen in water at 25°C were obtained from table 7.

	logarithmic normal (equation (5.6))	generalized Danckwerts (equation (5.5))
	wind speed = 2 m s^−1^	
*k*_L_ for hydrogen (m s^−1^)	1.14 × 10^−5^	1.32 × 10^−5^
*k*_L_ for oxygen (m s^−1^)	6.76 × 10^−6^	7.83 × 10^−6^
	wind speed = 4.2 m s^−1^	
*k*_L_ for hydrogen (m s^−1^)	2.73 × 10^−5^	2.90 × 10^−5^
*k*_L_ for oxygen (m s^−1^)	1.62 × 10^−5^	1.70 × 10^−5^
	wind speed = 8 m s^−1^	
*k*_L_ for hydrogen (m s^−1^)	3.91 × 10^−5^	4.10 × 10^−5^
*k*_L_ for oxygen (m s^−1^)	2.32 × 10^−5^	2.40 × 10^−5^

Although we have assumed steady-state conditions in this section, an unsteady-state version of the GD age distribution can also be derived whose details are not presented here. It is not obvious to the authors how to extend the LN age distribution to transient conditions.

## 6. Concluding remarks

This work is based on the primary assumption that the individual renewal frequency of eddies or elements at the surface of a turbulent liquid obeys a Boltzmann-type distribution. With a further assumption that the individual renewal frequency is either discrete or continuous, a population balance leads to the Danckwerts age distribution or a uniform age distribution, a special case of which is the Higbie age distribution. Thus, the Higbie and Danckwerts distributions are analogous to the Rayleigh–Jeans and Planck formulae, respectively, which describe the energy spectrum radiated by a blackbody.

The analysis of transient physical absorption of a gas into a large volume of liquid using the four age distributions revealed the crucial difference between the rate of absorption of the gas at the gas–liquid interface and the rate of its transfer to the bulk liquid. Under transient conditions, these two rates are not equal and have an inverse relationship. However, with the progress of absorption towards steady state, they approach one another.

The conventional, one-parameter Danckwerts age distribution can be generalized to a two-parameter age distribution which is able to capture the bell-shaped nature of the distribution of ages of surface elements that has been observed experimentally in air–sea heat and gas exchange. This distribution, called the GD age distribution, is equivalent to the two-parameter LN age distribution that has often been used by the physical oceanography community. Estimates of the liquid-side mass-transfer coefficient made using these two age distributions for the absorption of hydrogen and oxygen in water are very close to one another and are comparable to experimental values reported in the literature.

## Supplementary Material

The Generalized Danckwerts Age Distribution Function

## Data accessibility

All theoretical calculations presented herein were performed with the equations developed in this paper. The only experimental data referred to or used in this work are those in the prior manuscripts of Chung *et al.* [8], Garbe *et al*. [32] and Hutchinson & Sherwood [49].

## References

[1] Scherrer R 2006 Quantum mechanics: an accessible introduction. New York, NY: Addison-Wesley.

[2] Danckwerts PV 1951 Significance of liquid-film coefficients in gas absorption*.* Ind. Eng. Chem. (Eng. Process Dev.) 43, 1460–1467. (doi:10.1021/ie50498a055)

[3] Higbie R 1935 The rate of absorption of a pure gas into a still liquid during short periods of exposure. Am. Inst. Chem. Eng. 35, 365–389.

[4] Lamb WB, Springer TG, Pigford RL 1969 An interface impedance bridge. Ind. Eng. Chem. Fundam. 8, 823–827. (doi:10.1021/i160032a039)

[5] Johnson AI, Huang C-J 1956 Mass transfer studies in an agitated vessel. AIChE J. 2, 412–419. (doi:10.1002/aic.690020322)

[6] Hariott P 1962 A random eddy modification of the penetration theory. Chem. Eng. Sci. 17, 149–154. (doi:10.1016/0009-2509(62)80026-8)

[7] Lamont JC, Scott DS 1970 An eddy cell model of mass transfer into the surface of a turbulent liquid. AIChE J. 16, 513–519. (doi:10.1002/aic.690160403)

[8] Chung BTF, Fan LT, Hwang CL 1971 Surface renewal and penetration models in the transient state. AIChE J. 17, 154–160. (doi:10.1002/aic.69017013)

[9] Sada E, Katoh S, Yoshii H, Ban Y 1979 Rates of gas absorption with interfacial turbulence caused by micro-stirrers. Can. J. Chem. Eng. 57, 704–706. (doi:10.1002/cjce.545057060)

[10] Babu DR, Narsimhan G 1980 A surface renewal model for unsteady state transfer processes. Chem. Eng. J. 20, 169–175. (doi:10.1016/0300-9467(80)80001-3)

[11] Luk S, Lee YH 1986 Mass transfer in eddies close to air-water interface. AIChE J. 32, 1546–1554. (doi:10.1002/aic.690320915)

[12] Seo YG, Lee WK 1988 Single-eddy model for random surface renewal. Chem. Eng. Sci. 43, 1395–1402. (doi:10.1016/0009-2509(88)85112-1)

[13] Asher WE, Pankow JF 1991 Prediction of gas/water mass transport coefficients by a surface renewal model. Environ. Sci. Technol. 25, 1294–1300. (doi:10.1021/es00019a01)

[14] Fan LT, Shen BC, Chou ST 1993 The surface-renewal theory of interphase transport: a stochastic treatment. Chem. Eng. Sci. 48, 3971–3982. (doi:10.1016/0009-2509(93)80376-2)

[15] Maucci E, Briens CL, Martinuzzi RJ, Wild G 2001 Modeling of transient particle-liquid mass transfer in liquid and liquid-solid systems. Chem. Eng. Sci. 56, 4555–4570. (doi:10.1016/S0009-2509(00)00461-9)

[16] Lesage F, Midoux N, Latifi MA 2002 Momentum transfer in a fixed-bed reactor described by the surface renewal model. Chem. Eng. Sci. 57, 5115–5122. (doi:10.1016/S0009-2509(02)00426-8)

[17] Jajuee B, Margaritis A, Karamanev D, Bergougnou MA 2006 Application of surface-renewal-stretch model for interface mass transfer. Chem. Eng. Sci. 61, 3917–3929. (doi:10.1016/j.ces.2006.01.026)

[18] Kuthan K, Broz Z 1989 Mass transfer in liquid films during absorption. Part III. Dependence of the liquid-side mass transfer coefficient on the molecular diffusivity of gases at high values of the Schmidt number. Chem. Eng. Proc. 25, 75–84. (doi:10.1016/0255-2701(89)80033-9)

[19] Astarita G 1967 Mass transfer with chemical reaction. Amsterdam, The Netherlands: Elsevier.

[20] Komori S, Murakami Y, Ueda H 1989 The relationship between surface-renewal and bursting motions in an open-channel flow. J. Fluid Mech. 203, 103–123. (doi:10.1017/S0022112089001394)

[21] Banerjee S 2007 Modeling of interphase turbulent transport processes. Ind. Eng. Chem. Res. 46, 3063–3068. (doi:10.1021/ie061097)

[22] Turney D, Banerjee S 2008 Transport phenomena at interfaces between turbulent fluids. AIChE J. 54, 344–349. (doi:10.1002/aic.11427)

[23] Metzger I, Dobbins WE 1967 Role of fluid properties in gas transfer. Environ. Sci. Technol. 1, 57–65. (doi:10.1021/es60001a00) 22148339 10.1021/es60001a006

[24] Llaguno C, Muriel A 2010 Quantum turbulence at room temperature. J. Vac. Sci. Technol. A. 28, 306–308. (doi:10.1116/1.3294720)

[25] Jirkovsky L, Muriel A 2012 Pipe flow and wall turbulence using a modified Navier–Stokes equation. Commun. Theor. Phys. 57, 477–481. (doi:10.1088/0253-6102/57/3/22)

[26] Coulson JM, Richardson JF, Backhurst JR, Harker JH 1999 Coulson and Richardson's chemical engineering, vol. 1, 6th edn. Oxford, UK: Butterworth-Heinemann.

[27] Chatterjee SG 2010 A theoretical analysis of dissolved-gas transfer to the bulk liquid in gas absorption with a first-order reaction. Indian Chem. Eng. 51, 177–193. (doi:10.1080/00194500903315476)

[28] Danckwerts PV 1970 Gas-liquid reactions. New York, NY: McGraw-Hill.

[29] Loffler DG, Merchuk JC 1972 Steady and unsteady state absorption with first order chemical reaction in a CFSTR, evaluated with film-penetration model. Lat. Am. J. Chem. Eng. Appl. Chem. 2, 175–183.

[30] Merchuk JC 1985 On the use of the film model for analysis of gas-liquid reactions in CSTR. Chem. Eng. Commun. 39, 389–390. (doi:10.1080/00986448508911685)

[31] Westerterp KR, van Swaaij WPM, Beenackers AAC 1984 Chemical reactor design and operation. Amsterdam, The Netherlands: John Wiley & Sons.

[32] Garbe CS, Jähne B, Haußecker H 2002 Measuring the sea surface heat flux and probability distribution of surface renewal events. In Gas transfer at water surface (eds Donelan MA, Drennan WM, Saltzman ES, Wanninkhof R), pp. 109–114. Washington, DC, USA: American Geophysical Union.

[33] Garbe CS, Schimpf U, Jähne B 2004 A surface renewal model to analyze infrared image sequences of the ocean surface for the study of air-sea heat and gas exchange. J. Geophys. Res. 109, C08S15 (doi:10.1029/2003JC001802)

[34] Veron F, Melville WK, Lenain L 2011 The effects of small-scale turbulence on air–sea heat flux. J. Phys. Oceanogr. 41, 205–220. (doi:10.1175/2010JPO4491.1)

[35] Chatterjee SG 2013 Two contrasting paradigms of science: Cartesian mechanism and undivided wholeness. Int. J. Sci. Soc. 4, 75–93.

[36] Koltuniewicz A, Noworyta A 1994 Dynamic properties of ultrafiltration systems in light of the surface renewal theory. Ind. Eng. Chem. Res. 33, 1771–1779. (doi:10.1021/ie00031a01)

[37] Koltuniewicz A, Noworyta A 1995 Method of yield evaluation for pressure-driven membrane processes. Chem. Eng. J. 58, 175–182. (doi:10.1016/0923-0467(95)02981-8)

[38] Chatterjee SG 2010 On the use of the surface-renewal concept to describe cross-flow ultrafiltration. Indian Chem. Eng. 52, 179–193. (doi:10.1080/00194506.2010.54792)

[39] Hasan A, Peluso CR, Hull TS, Fieschko J, Chatterjee SG 2013 A surface-renewal model of cross-flow microfiltration. Braz. J. Chem. Eng. 30, 167–186. (doi:10.1590/S0104-66322013000100019)

[40] Jiang S, Chatterjee SG 2015 A surface-renewal model for constant flux cross-flow microfiltration. J. Appl. Polym. Sci. 132, 41778 (doi:10.1002/app.4177)

[41] Zhang W, Chatterjee SG 2015 Influence of residence-time distribution on a surface-renewal model of constant-pressure cross-flow microfiltration. Braz. J. Chem. Eng. 32, 139–154. (doi:10.1590/0104-6632.20150321s00003129)

[42] Idan G, Chatterjee SG 2015 Application of a surface-renewal model to permeate-flux data for constant-pressure cross-flow microfiltration with Dean vortices. Braz. J. Chem. Eng. 32, 609–627. (doi:10.1590/0104-6632.20150322s00003417)

[43] Mondal C, Das A, Chatterjee SG 2016 A time-lag model for biogas production by anaerobic digestion. J. Renew. Sustain. Energy 8, 063104 (doi:10.1063/1.4966160)

[44] Das A, Mondal C, Chatterjee SG 2016 Time-lag models for continuous stirred tank and plug flow digesters for biogas production. Energy Fuels 30, 10 404–10 416. (doi:10.1021/acs.energyfuels.6b01320)

[45] Méndez V, Fort J, Farjas J 1999 Speed of wave-front solutions to hyperbolic reaction-diffusion equations. Phys. Rev. E 60, 5231–5243. (doi:10.1103/PhysRevE.60.5231) 10.1103/physreve.60.5231 11970393

[46] Fort J, Méndez V 2002 Wavefronts in time-delayed reaction-diffusion systems. Theory and comparison to experiment. Rep. Prog. Phys. 65, 895–954. (doi:10.1088/0034-4885/65/6/201)

[47] Lema MA, Golombek DA, Echave J 2000 Delay model of the circadian pacemaker. J. Theor. Biol. 204, 565–573. (doi:10.1006/jtbi.2000.2038) 10833356 10.1006/jtbi.2000.2038

[48] Bullin JA, Dukler AE 1972 Random eddy models for surface renewal: formulation as a stochastic process. Chem. Eng. Sci. 27, 39–442. (doi:10.1016/0009-2509(72)85081-4)

[49] Hutchinson MH, Sherwood TK 1937 Liquid film in gas absorption. Ind. Eng. Chem. 29, 836–840. (doi:10.1021/ie50331a023)

[50] Perry RH, Green DW, Maloney JO (eds). 1984 Perry’s chemical engineers' Handbook, 6th edn New York, NY: McGraw-Hill.

