# Supplementary material for: A frequency quantum interpretation of the surface renewal model of mass transfer
Source: R Soc Open Sci. 2017 Jul 5;4(7):170103. doi: 10.1098/rsos.170103 (PMC5541532; doi:10.1098/rsos.170103)
Supplement: The Generalized Danckwerts Age Distribution Function [file rsos170103supp1.pdf]

APPENDIX. The Generalized Danckwerts age distribution[1]

In this appendix, we indicate a derivation and provide a discussion of the generalized Danckwerts age distribution. For simplicity, the analysis will be restricted to steady state conditions (i.e., $t_p \to \infty$).

The differential equation that describes the steady state conventional Danckwerts age distribution is given by [see equation (2.7)]

$$\frac{df}{dt} + Sf = 0. \tag{A.1}$$

We now introduce a time delay into equation (A.1) by assuming that the eddy renewal process, represented by the term Sf, does not instantaneously affect the time rate of change of f (i.e., df/dt), but affects it only τ_d units of time later. Equation (A.1) can then be expressed as

$$\left(\frac{df}{dt}\right)_{t+\tau_d} + Sf = 0. \tag{A.2}$$

Expanding the derivative in equation (A.2) in a Taylor series and retaining only the first two terms of this series transforms this equation into the following second-order differential equation:

$$\tau_d \frac{d^2 f}{dt^2} + \frac{df}{dt} + Sf = 0, \tag{A.3}$$

whose characteristic equation is

$$\tau_d p^2 + p + S = 0, \tag{A.4}$$

[1] This appendix presents a derivation of the generalized Danckwerts age distribution function used in the manuscript entitled “A frequency quantum interpretation of the surface renewal model of mass transfer” (authors: C. Mondal and S. G. Chatterjee), which has been accepted for publication in Royal Society Open Science.

where p is a root of equation (A.4). The case of real and equal roots of this equation occurs for $\tau_d = 0.25/S$, i.e., the greater the value of the fundamental renewal frequency S, the smaller is the lag time τ_d. For this case, the general solution of equation (A.3) is given by

$$f(t) = (K_1 + K_2 t)e^{-2St}, \tag{A.5}$$

where K_1 and K_2 are constants. Using the condition $f(0) = 0$ in equation (A.5) yields K_1 = 0, thereby reducing it to

$$f(t) = K_2 t e^{-2St}. \tag{A.6}$$

Differentiation of this equation gives

$$\frac{df}{dt} + Sf\left(2 - \frac{1}{St}\right) = 0, \tag{A.7}$$

which can be compared to equation (A.1).

Equation (A.7) suggests the following general differential equation for f:

$$\frac{df}{dt} + Sf\left(n - \frac{a}{St}\right) = 0, \tag{A.8}$$

where a and n are parameters. An examination of this equation reveals that for $t < a/(nS)$, $df/dt > 0$ whereas for $t > a/(nS)$, $df/dt < 0$. Thus, the curve of f versus t passes through a maximum. The solution of equation (A.8) is given by

$$f(t) = Kt^a e^{-nSt}, \tag{A.9}$$

where K is a constant. Also

$$\int_0^\infty f(t)dt = 1. \tag{A.10}$$

Substituting equation (A.9) into equation (A.10) yields

$$K = \frac{(nS)^{a+1}}{\Gamma(a+1)}\,, \tag{A.11}$$

which upon substitution into equation (A.9) transforms it into

$$f(t) = \frac{(nS)^{a+1}}{\Gamma(a+1)} t^a e^{-nSt}. \tag{A.12}$$

A relation between n and a can be obtained by performing a mass balance for dissolved gas in the liquid. For physical absorption, this balance states that, under steady state conditions, the rates of gas absorption and dissolved gas transfer to the bulk liquid should be equal. These two rates are given by

$$R_{abs} = \int_0^\infty R_{inst}(t)\, f(t)dt \tag{A.13}$$

and

$$R_{trans} = S\int_0^\infty \int_0^\infty [c(x,t) - c_b]\, f(t)dxdt. \tag{A.14}$$

By using equations (3.1), (3.2) and (A.12) in equations (A.13) and (A.14) and by equating R_{abs} and R_{trans}, it can be shown that

$$n = 2a + 1 \tag{A.15}$$

and (at steady state)

$$R_{abs} = R_{trans} = \alpha\sqrt{DS}(c_s - c_b) = k_L(c_s - c_b), \tag{A.16}$$

where $k_L = \alpha\sqrt{DS}$ with

$$\alpha = \frac{\Gamma\left(a + {}^{1}\!/_{2}\right)}{\Gamma(a+1)}\sqrt{\frac{2a+1}{\pi}}\,. \tag{A.17}$$

Upon substitution of equation (A.15) into equation (A.12), we finally obtain the generalized Danckwerts age distribution:

$$f(t) = S\frac{(2a+1)^{a+1}}{\Gamma(a+1)}(St)^a e^{-(2a+1)St}. \tag{A.18}$$

For a = 0, equation (A.18) reduces to $f(t) = Se^{-St}$, which is the conventional Danckwerts age distribution. Equation (A.18) can be expressed in dimensionless form as

$$f^*(t^*) = \frac{f(t)}{S} = \frac{(2a+1)^{a+1}}{\Gamma(a+1)}(t^*)^a e^{-(2a+1)t^*}, \tag{A.19}$$

where $t^* = St$. From equation (A.19) it is seen that $f^*(0) = f^*(\infty) = 0$ and thus the curve of $f^*(t^*)$ versus t^* passes through a maximum (as was anticipated earlier), which occurs at $t^* = t^*_{crit}$. The value of t^*_{crit} can be obtained by setting $df^*/dt^* = 0$ using equation (A.8) and is given by

$$t^*_{crit} = St_{crit} = \frac{a}{2a+1}\,, \tag{A.20}$$

where t_{crit} is the value of the age where f is a maximum. The maximum or critical value of f^* can be obtained by substituting equation (A.20) into equation (A.19), which yields

$$f^*_{crit} = f^*(t^*_{crit}) = \frac{(2a+1)^{a+1}}{\Gamma(a+1)} \left(\frac{a}{2a+1}\right)^a e^{-a}. \qquad (A.21)$$

Figure A1 shows the behavior of $f^*(t^*)$ as a function of t^* and Table A1 reports values of t^*_{crit}, f^*_{crit} and α for values of a = 0, 1, 2, and 3. As the parameter a increases, $t^*_{crit} \rightarrow 0.5$ and the dimensionless age distribution becomes increasingly narrower.

Table A1. Values of t^*_{crit}, f^*_{crit} and α calculated from equations (A.20), (A.21) and (A.17), respectively, for different values of the parameter a of the dimensionless generalized Danckwerts age distribution [equation (A.19)].

a	t^*_{crit}	f^*_{crit}	α
0	0	1	1
1	1/3 (≈ 0.3333)	3/e (≈ 1.1036)	$\sqrt{3}/2$ (≈ 0.8660)
2	2/5 (= 0.4)	10/e^2 (≈ 1.3534)	$3\sqrt{5}/8$ (≈ 0.8385)
3	3/7 (≈ 0.4286)	31.5/e^3 (≈ 1.5683)	$5\sqrt{7}/16$ (≈ 0.8268)

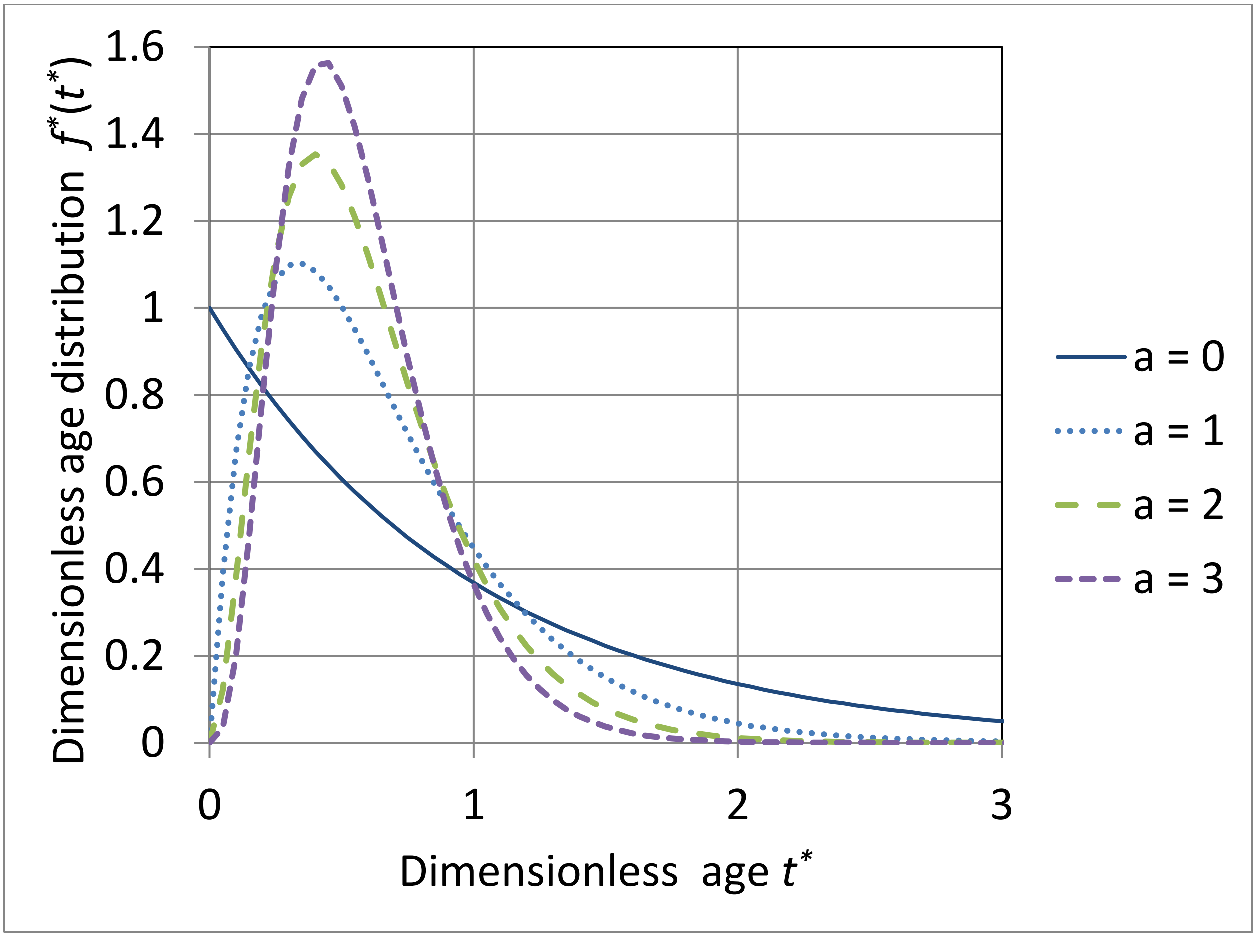

Figure A1. Plot of the generalized Danckwerts age distribution [equation (A.19)] in dimensionless coordinates.
